# Natural *versus* organophilized smectites as drug adsorbents: experiment and molecular modeling

**DOI:** 10.1039/d5ra04769b

**Published:** 2025-09-30

**Authors:** Jonáš Tokarský, Pavlína Peikertová, Klára Výšková, Markéta Davidová, Silvie Vallová

**Affiliations:** a Department of Thermal Engineering, Faculty of Materials Science and Technology, VSB-Technical University of Ostrava 17. listopadu 15/2172, Ostrava-Poruba 708 00 Czech Republic; b Nanotechnology Centre, CEET, VSB-Technical University of Ostrava 17. listopadu 2172/15, 708 00 Ostrava-Poruba Czech Republic; c Department of Chemistry and Physico-Chemical Processes, Faculty of Materials Science and Technology, VSB-Technical University of Ostrava 17. listopadu 15/2172, Ostrava-Poruba 708 00 Czech Republic silvie.vallova@vsb.cz

## Abstract

To adsorb pollutants from water, smectites are commonly modified with quaternary ammonium compounds. However, these are also environmentally hazardous. This study aims to highlight that original smectites can compete with modified ones and that modification is not necessary. Original smectites – montmorillonite (MMT), beidellite (BEI), nontronite (NON) – and the same smectites modified with tetradecyltrimethylammonium (TTA^+^), denoted as MMT-M, BEI-M, NON-M, were studied as adsorbents of drugs ampicillin (AMP) and lamotrigine (LAM). Adsorbents before and after adsorption were studied using Fourier-transform infrared spectroscopy, elemental analysis, X-ray powder diffraction, thermogravimetry, and molecular modeling. The adsorption efficiency of original smectites reaches or exceeds (for LAM on BEI and NON) 50 mg per 1 g of adsorbent. Adsorption is not monolayer and the order BEI > MMT > NON for both AMP and LAM was found. While AMP is preferentially adsorbed through interaction with Na^+^, LAM is preferentially adsorbed through interactions with other LAMs. For modified smectites, the adsorption efficiency ranges from units to lower tens of mg per 1 g of adsorbent in the order MMT-M > NON-M > BEI-M and NON-M > MMT-M > BEI-M for AMP and LAM, respectively. The adsorption can be considered monolayer, and it is not controlled only by the strength of drug–TTA^+^ interaction. It can be concluded that (1) the modification did not enhance the adsorption efficiency of original smectites, (2) the original smectites showed higher adsorption efficiency compared to the modified ones, and (3) the original smectites are a suitable environmentally friendly alternative to the commonly used modified smectites.

## Abstract 2

Original smectites exhibit the same or higher adsorption efficiency of drugs (ampicillin and lamotrigine) from water compared to smectites organophilized with environmentally hazardous tetradecyltrimethylammonium cations.

## Graphical abstract

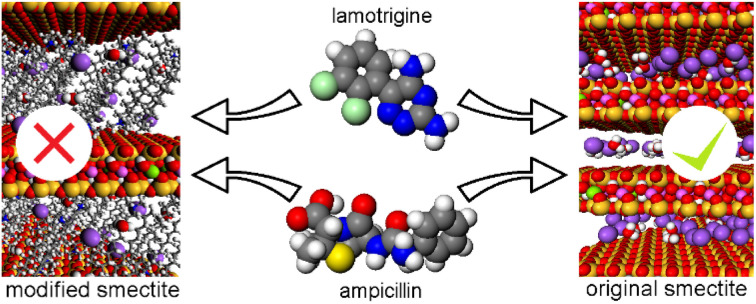

## 1 Introduction

Pharmaceuticals in wastewater and natural waters pose an environmental risk, not only because of their penetration into drinking water,^1–4^ but also due to the impact on aquatic and terrestrial flora and fauna,^5–10^ while the long-term effect on the environment and human health is still a subject of research.^1^

Adsorption is an effective way to remove pharmaceuticals from water.^2,5,9^ Materials suitable for this purpose include layered clay minerals (phyllosilicates), especially smectites^2,5,6,9,11–15^ exhibiting all the necessary properties of a good adsorbent: abundance, cheapness, chemical stability, and large surface area.^2,5,9,11,12,16^ The layered structure together with the negative charge is responsible for the ability to draw cations and polar compounds into the interlayer space. This process is called intercalation.^17–19^ Smectite layers are of the 2 : 1 type, *i.e.* two tetrahedral sheets bonded to one octahedral sheet sandwiched between them form a single layer of phyllosilicate. Depending on the charge of the dominant metal ion M^*X*+^ in the octahedral sheet, either all possible positions (when *X* = 2, *e.g.* Mg^2+^) or only two thirds of the possible positions (when *X* = 3, *e.g.* Al^3+^) are occupied. The sheet (and the layer as well as the phyllosilicate) is accordingly termed trioctahedral and dioctahedral, respectively.^20^ Montmorillonite, beidellite and nontronite are dioctahedral smectites, whereas hectorite, saponite and stevensite are trioctahedral smectites.^21,22^

Before their use as adsorbents, smectites are often modified (organophilized) by intercalation of cationic surfactants. Surfactants used include quaternary ammonium compounds (QACs), *e.g.* hexadecyltrimethyl ammonium,^23–27^ tetradecyltrimethyl ammonium,^15,25,26,28,29^ or dodecyldimethylbenzyl ammonium,^2,23^ to name a few. Modification with QACs increases the basal distance of smectites facilitating the entry of adsorbate into the interlayer space and increasing the adsorption efficiency, as is often emphasized in adsorption studies.^2,15,25,27,29^ However, what the reader does not usually find in adsorption studies is the environmental risk of the QACs themselves. One has to look at studies from other research areas to find that dodecyl-, tetradecyl-, and hexadecyltrimethylammonium were found toxic to aquatic organisms^30–32^ or that hexadecyl- and octadecyltrimethyl ammonium were found phytotoxic^33^ or toxic to soil bacteria.^34,35^ For more information, the reader is also referred to the records of these QACs in the PubChem database.^36–41^ This environmental aspect is commonly neglected in adsorption studies highlighting the modifed clays (even described as “environmental adsorption materials”^26^ or “environmentally friendly adsorbent”^27^), and the possibility of using original smectites as an alternative is only rarely explicitly mentioned.^23^ Although non-toxic surfactants can be sought and tested,^23^ the use of original smectites is advantageous because no organophilization means savings in chemicals, energy, time, *etc.*

Keeping the above facts in mind, we started our research focused on the adsorption of drugs from an aqueous environment onto three smectites, montmorillonite (MMT), beidellite (BEI), nontronite (NON), both natural and modified with tetradecyltrimethylammonium bromide (TTAB). Two drugs were chosen for the adsorption, namely ampicillin (a broad-spectrum antibiotic),^42^ and lamotrigine (an antidepressant and antiepileptic agent),^43^ which are often found in wastewater around the world.^4,7,10,44–52^ There are very few studies focused on the removal of ampicillin or lamotrigine from water using original smectites or smectite-based adsorbents. Ampicillin was adsorbed onto MMT,^2^ MMT modified with dodecyldimethylbenzylammonium,^9^ and MMT-rich bentonite decorated with Fe/Ni nanoparticles.^8^ In the case of lamotrigine, adsorption on MMT/polyvinylpyrrolidone composite was reported.^53^ For larger series of different original and QACs-modified smectites, a comparison of their adsorption efficiencies in the removal of drugs from water is lacking in the literature. Adsorption efficiencies of both original and QACs-modified forms can be found for MMT (with ampicillin^54^ or phenol^25^ as adsorbate), but not for BEI or NON. Such a comparison, however, is important to determine whether environmentally friendly natural smectites can be used as a suitable alternative to modified smectites. Moreover, comparison of results from various studies is complicated by different experimental conditions, *e.g.*, initial drug concentration, volume of solution used, amount of adsorbent added, duration of adsorption, *etc.*

The novelty of this study is that the adsorption of two different drugs on three different smectites, each original and QACs-modified (*i.e.*, twelve systems) was performed under exactly the same conditions. The results are therefore directly comparable without the above-mentioned complications. Considering eight different concentrations of each drug, this study is based on a total of ninety-six samples. To our knowledge, such a set of data obtained under the same conditions is not yet available in the literature focused on for drug adsorption on original and QAC-modified smectites.

A force field-based molecular modeling in close collaboration with instrumental analytical methods was used to investigate the structures before and after adsorption of ampicillin or lamotrigine with the aim of revealing the influence of layer charge and the modification. In molecular models, in addition to the interactions of drug molecules with the adsorbent, we also examined the drug–drug interactions, which are not always given due attention, although their role may not be negligible.

The main goal of our study is to determine whether environmentally friendly natural smectites can be used as a suitable alternative to modified smectites.

## 2 Materials and methods

### 2.1 Materials

The original natural smectites MMT (STx-1, Texas, USA),^55^ BEI (SBId-1, Idaho, USA),^55^ and NON (NAu-1, South Australia)^55^ were provided by the Institute of Geonics of the CAS. Chemical compositions of the samples are provided in Table S1. For the original MMT, BEI, and NON (100 mg each) in deionized water (20 cm^3^), the pH value was 7.2, 6.7, and 7.0, respectively. Ethanol (97%) was purchased from MACH CHEMIKÁLIE s.r.o. (Czech Republic). Tetradecyltrimethylamonnium bromide (TTAB; CH_3_(CH_2_)_13_N^+^(CH_3_)_3_Br^−^; *M*_TTAB_ = 336.39 g mol^−1^) was supplemented by Carl Roth GmbH (Germany), while the ampicillin (AMP; C_16_H_19_N_3_O_4_S; CAS no. 69-53-4; *M*_AMP_ = 349.40 g mol^−1^; p*K*_a_ = 2.65)^56^ and lamotrigine (LAM; C_9_H_7_Cl_2_N_5_; *M*_LAM_ = 256.09 g mol^−1^; p*K*_a_ = 5.70)^57^ were purchased from Sigma-Aldrich (USA). For AMP and LAM in deionized water (400 mg dm^−3^), the pH value was 5.9 and 7.1, respectively. Deionized water was used for adsorption experiments. A scheme showing the processing of smectites and the subsequent sequence of experiments and analyses is provided in Fig. S1.

### 2.2 Methods

#### 2.2.1 Modification of smectites

For each smectite (MMT, BEI, NON), 1 g of the mineral with 20 cm^3^ of TTAB aqueous solution (*c*_TTAB_ = 0.1 mol dm^−3^; *i.e. m*_TTAB_ = 0.67278 g and *m*_TTA^+^_ = 0.51298 g) were shaken in a rotary shaker Hei-MIX Reax 2 (Heidolph Instruments, Germany) for 3.5 h. Then, the suspensions were centrifuged using centrifuge EBA 12 (Hettich, Germany) at 6000 rpm for 20 min to separate solid and liquid phase. To the solid phase, 10 cm^−3^ of ethanol : water mixture (1 : 1 volume ratio) was added, and the samples were centrifuged again for 10 min. This procedure was repeated three times. Finally, 10 cm^3^ of ethanol (97%) was added to the separated solid phase and centrifuged for 10 min. The separated solid fraction was dried at 25 °C in a laboratory. Such prepared samples of modified MMT, BEI, and NON were denoted as MMT-M, BEI-M, and NON-M, respectively. For the MMT-M, BEI-M, and NON-M (100 mg each) in deionized water (20 cm^3^), the pH value was 6.7, 6.3, and 6.5, respectively.

#### 2.2.2 Characterization methods

X-ray fluorescence spectroscopy (XRFS) analysis of powder samples compressed into tablets (with wax as a binder) was perfomed on a energy dispersive fluorescence spectrometer SPECTRO XEPOS (SPECTRO Analytical Instruments GmbH, Kleve, Germany) equipped with a Pd X-ray tube with power of 50 W.

Fourier-transform infrared (FTIR) spectra were recorded in the range of 400–4000 cm^−1^ by Nicolet iS50 – Thermo Fisher Scientific with diamond ATR crystal (spectral resolution 4 cm^−1^, 32 scans). The elemental analysis was performed using Elementar Vario EL Cube analyser (Elementar, Germany). Accuracy of the analysis (<0.1 wt% for each element: C, H, N, S) was ensured by simultaneous analysis of 4-aminobenzenesulfonic acid (5 mg) as a standard.

The X-ray powder diffraction (XRPD) analysis was performed on a Rigaku MiniFlex Theta/2Theta powder diffractometer (Rigaku, Japan) equipped with a D/theX ultra detector with an Fe foil serving as a beta filter. The source of a primary X-ray beam was a Co lamp (*λ*_Co(Kα)_ = 1.7889 Å). Current of 15 mA and voltage of 40 kV were used.

The thermogravimetry analysis (TGA) was carried out on a simultaneous thermal analyzer SDT 650 (TA Instruments, USA) with horizontal dual-beam design for heat flow and weight measurements. Each sample (∼20 mg) in α-Al_2_O_3_ crucible was heated up to 1000 °C (10 °C min^−1^) in a dynamic (100 cm^3^ min^−1^) air atmosphere. Mass ratios of TTA^+^ (*w*_TTA^+^_TGA_; wt%) in the modified smectites were calculated according to the equation (eqn (1))^58^

1 *w*_TTA^+^_TGA_ = 100 × (Δ*m*_mod.smect._ − Δ*m*_smect._)/(Δ*m*_TTAB_ − Δ*m*_smect._)

where Δ*m*_mod.smect._, Δ*m*_smect._, and Δ*m*_TTAB_ is a mass loss (in wt%) of a modified smectite, original smectite, and pure TTAB, respectively. Each mass loss was taken from the same temperature range (150–985 °C).

The pH measurements were performed on a pH50 instrument (XS Instruments, Carpi, Italy) equipped with 201T-F all-in-one pH/ttemp. electrode (Apera Instruments, Columbus, Ohio, USA). All pH measurements were performed at 25 °C.

The high-perfomance liquid chromatography (HPLC) was performed on Nexera X2 (Shimadzu, Japonsko) chromatograph coupled to a QTRAP 6500+ mass spectrometer (MS; Sciex, USA) and an ESI + ionization source. A 150 mm long phenyl-hexyl column with an internal diameter of 3 mm and a mobile phase consisting of ammonium formate in water and methanol was used to separate the individual components. Capillary voltage and temperature was 5.5 kV and 450 °C, respectively. Nebulizer gas and heater gas pressure was 344.74 and 413.69 kPa, respectively. Based on the equilibrium concentrations of drugs in solutions obtained from the HPLC-MS analysis, the adsorption capacity (*q*_e_; mg g^−1^) was determined according to the equation (eqn (2))

2 *q*_e_ = *V* × (*c*_0_ − *c*_r_)/*m*

where *V* (dm^3^) is volume of the solution used, *c*_0_ (mg dm^−3^) is the original drug concentration, *c*_r_ (mg dm^−3^) is the drug concentration at the equilibrium, and *m* (g) is the mass of the adsorbent.

Experimental data were supplemented with the results of force field-based molecular modeling performed in the Materials Studio 4.2 (MS; Biovia company, CA, USA) modeling environment. Models of TTA^+^ ions, water and drug molecules were built in MS/Visualizer sketching tool. Periodic unit cell with lattice parameters *a* = 5.21 Å, *b* = 9.02 Å, *c* = 15.00 Å, *α* = *γ* = 90°, *β* = 95.18°^59^ was used in the MS/Crystal Builder module to create 7*a* × 2*b* × 1*c* supercells having the crystallochemical formula (Al_42_Mg_12_Fe_2_^3+^)(Si_112_)O_280_(OH)_56_ (MMT), (Al_51_Fe_3_^3+^MgTi)(Si_105_Al_7_)O_280_(OH)_56_ (BEI), and (Al_8_Fe_47_Mg)(Si_98_Al_14_)O_280_(OH)_56_ (NON) with the layer charge of −12, −7, and −15, respectively. These models were used to study the composition and space arrangement of the interlayer content. The crystallochemical formulas were determined according to Deer *et al.*^60^ from XRFS data in correlation with the Physical and Chemical data of Source Clays.^55^ For unit cell compositions and additional information, the reader is referred to SI material.

To study the surfaces, each supercell was cleaved along the (001) plane, creating a model of the given surface (periodic in the direction of the *a* and *b* axes) which was completed by adding a 400 Å high vacuum slab (in the direction perpendicular to the surface).^61^

Universal force field (UFF)^62^ was applied because it is able to parameterize atoms in both inorganic and organic components and has been already successfully used for this type of organo-/inorganic hybrid structures.^63–67^ Since the UFF does not contain intrinsic atomic charges, the charges in smectites and molecules were calculated separately. Charge equilibration (QEq)^68^ and Gasteiger^69^ method, respectively, were used, namely the QEq_charged1.1 set (suitable for silicates) and the Gast_polygraf1.0 set (suitable for organic molecules including those with tetravalent nitrogen) as implemented in the MS. The negative layer charges −12 (MMT), −5 (BEI), and −15 (NON) were compensated by Na^+^ and TTA^+^ cations (of different ratios). Water molecules were also added in varying amounts.

In the models of interlayer, Na^+^ and TTA^+^ cations were placed together into the interlayer space. Dozens of initial models were prepared containing different ratios of Na^+^ and TTA^+^ and different amounts of water molecules. Each initial model with a given composition was prepared in many variants with various initial space arrangements of the molecules. For the subsequent preparation of models of modified smectites with drug molecules in the interlayer, only models with the lowest total potential energy and *d*_001_ values corresponding to the *d*_001_ values of real modified smectites were used.

In the models of surface, various amounts of TTA^+^ were placed on one side of the surface (1, 7, or all, *i.e.* 12, 5, and 15 for MMT, BEI, and NON, respectively). On the opposite side of the surface, such a number of Na^+^ was placed that the layer charge was fully compensated. In the models of interlayer space, drug molecules were placed into the interlayer space. In the models of surface, drug molecules were placed on the same side as TTA^+^. Also, in the case of these twelve studied systems – two drugs on three original or three modified smectites – dozens of initial models were prepared containing selected ratios of Na^+^ and TTA^+^, different amounts of water molecules and one or more drug molecules (more drug molecules in the case of models of original smectites without TTA^+^). Each initial model with a given composition was prepared in many variants with various initial space arrangements of the molecules. In the case of the interlayer of each studied system, five optimized models with both the lowest total potential energy and *d*_001_ values corresponding to the *d*_001_ values of real samples were accepted as representative for the given system. In the case of surfaces where the *d*_001_ value is meaningless, five optimized models with the lowest energies were accepted for each system.

Due to the large number of initial models in this study, geometry optimization was chosen instead of molecular dynamics. Phyllosilicate-based organo-/inorganic structures have previously been studied using geometry optimization, and the results were found reliable,^63,65,66^ although this approach may not represent a complete exploration of possible conformations, as molecular dynamics does.

The geometry optimization was performed using the UFF and Smart algorithm in MS/Forcite module. The following convergence criteria were used: Δ*d* = 1.5 × 10^−2^ Å, Δ*E* = 1 × 10^−3^ kcal mol^−1^, and Δ*F* = 0.5 kcal mol^−1^ Å^−1^. The external pressure and number of iteration steps were set to 101 325 Pa and 5 × 10^5^, respectively. Van de Waals cutoff distance was 12.5 Å. The rigidity of 2 : 1 layers was ensured by fixed parameters *a*, *b*, and *γ* during the geometry optimization.^70,71^ Basal spacings of the optimized models of the interlayer were determined in MS/Reflex module under conditions corresponding to real XRPD analyses (*λ* = 1.7889 Å, Bragg–Brentano geometry).

The drug–substrate interaction energy *E*_int_ (kcal mol^−1^) was calculated from the optimized models using the equation (eqn (3))

3 *E*_int_ = *E*_tot_ − (*E*_drug_ + *E*_w/drug_)

where *E*_tot_ (kcal mol^−1^) is a total potential energy of the whole optimized model, *E*_drug_ (kcal mol^−1^) is a total potential energy of the drug molecule (AMP or LAM), and *E*_w/drug_ (kcal mol^−1^) is a total potential energy of the optimized model without the drug molecule. The more negative the *E*_int_ value, the stronger the interaction. Other interaction energies were calculated analogously from selected parts of the optimized models. For more information, the reader is referred to the SI material.

#### 2.2.3 Adsorption experiments

In a typical experiment, 100 mg of a given modified smectite (*i.e.* MMT-M, BEI-M, or NON-M) or original smectite (*i.e.* MMT, BEI, or NON) was added to the aqueous solution (20 cm^3^) of a given drug (*i.e.* AMP or LAM). Eight different solutions with concentrations of 20, 40, 60, 80, 100, 200, 300, and 400 mg dm^−3^ were used. After 24 h of shaking the solution with the modifed smectite on a rotary shaker, the liquid fraction was separated from the solid fraction by filtration. The liquid fraction was subsequently analyzed by HPLC.

Adsorption equilibrium data were fitted by various model adsorption isotherms. In the case of the Freundlich adsorption isotherm (FAI),^72,73^ the following equation (eqn (4)) was used

4 *q*_e_ = *K*_F_ × *c*_e_^1/*n*^

where *q*_e_ (mg g^−1^) is an equilibrium adsorbed amount of drug at a given concentration, *n* (−) and *K*_F_ ((mg g^−1^) (dm^3^ g^−1^)^*n*^) are the isotherm constants, and *c*_e_ (mg dm^−3^) is the equilibrium concentration of drug in a solution.

In the case of the Langmuir adsorption isotherm (LAI),^73,74^ the following equation (eqn (5)) was used

5 *q*_e_ = *q*_m_ × *K*_L_ × *c*_e_/(1 + *K*_L_ × *c*_e_)

where *q*_m_ (mg g^−1^) is the maximum amount of drug required to cover the monolayer, and *K*_L_ (dm^3^ mg^−1^) is the isotherm constant.

In the case of the Toth adsorption isotherm (TAI),^73,75,76^ the following equation (eqn (6)) was used

6 *q*_e_ = *q*_m_ × *K*_T_ × *c*_e_/(1 + (*K*_T_ × *c*_e_)^*n*^) ^1/*n*^

*n* (−) and *K*_T_ (dm^3^ mg^−1^) are the isotherm constants.

The reproducibility of adsorption *r*(*q*_e_) (%) was quantified using the equation (eqn (7))

7 *r*(*q*_e_) = 100 × (Σ_*n*_ (*q*_e(1),*n*_/*q*_e(2),*n*_))/*n*

where *q*_e(1),*n*_ (mg g^−1^) is an equilibrium adsorbed amount of drug at a given (*n*-th) concentration in the first adsorption experiment, *q*_e(2),*n*_ (mg g^−1^) is an equilibrium adsorbed amount of drug at a given (*n*-th) concentration in the second adsorption experiment, Σ_*n*_ is the sum of all *n* ratios *q*_e(1),*n*_/*q*_e(2),*n*_, and *n* is the number of concentrations tested (specifically 8).

## 3 Results and discussion

### 3.1 Composition and structure of modified smectites

**Fig. 1:**
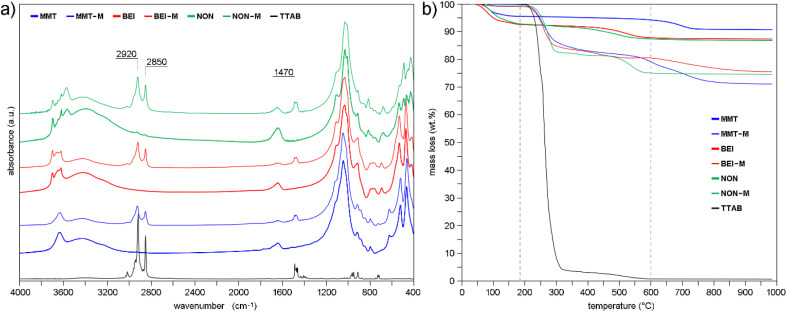
(a) FTIR spectra and (b) TGA curves of original smectites, modified smectites and TTAB.

FTIR spectra of the original and modified smectites (Fig. 1a) can be divided into the following regions: Si–O and Si–O–M vibrations (where M = Al, Fe, Mg) (400–700 cm^−1^), Al–O–H and (Al, Mg) –O–H vibrations (700–950 cm^−1^), Si–O and Si–O–Si vibrations (950–1200 cm^−1^), and O–H, Al–O–H and (Mg, Al)–OH vibrations (∼1640 cm^−1^ and 3100–3600 cm^−1^).^77^ See also Table S2.

Main bands of TTAB (Fig. 1a) are attributed to the symmetric and asymmetric stretching vibrations of alkyl chains (2921 and 2852 cm^−1^), symmetric and asymmetric stretching of C–H (∼1470 cm^−1^), the C–N^+^ stretching (964 cm^−1^), C–H *trans* out-of-plane bending vibration (913 cm^−1^) and C–H *cis* out-of-plane bending vibration (∼720 cm^−1^).^78,79^ The clearly distinguishable bands ∼2920, ∼2850 and ∼1470 cm^−1^ (Fig. 1a) prove the presence of TTA^+^ in the modified smectites.

The larger mass losses of the modified smectites compared to the original smectites, as detected by TGA (Fig. 1b), also demonstrate the presence of TTA^+^. The temperature interval of degradation of pure TTAB, indicated by vertical dashed lines (185 °C, 600 °C; Fig. 1b), was used to determine the amount of TTA^+^ in the modified samples. According to the eqn (1), MMT-M, BEI-M, and NON-M contains 19.8 wt%, 14.5 wt%, and 20.5 wt% of the TTA^+^, respectively. As expected, the TTA^+^ content increases with increasing layer charge of smectites.

Elemental analysis of the modified smectites (Table 1) agrese well TGA; the TGA analysis leads to only a slight overestimation of the TTA^+^ content. The amount of TTA^+^ determined as the sum of C + H + N (*w*_TTA^+^_EA_; Table 1) differs from the amount determined from TGA (*w*_TTA^+^_TGA_; Table 1) by an average of 1.78 ± 0.06 wt%. The origin of N and C from TTA^+^ is proved by the *w*_N_ : *w*_C_ ratio. While the ideal *w*_N_ : *w*_C_ for the TTA^+^ is 0.0686, the average *w*_N_ : *w*_C_ value obtained from the data in Table 1 is 0.0698 ± 0.0008.

**Table 1:** Elemental analysis (in wt%) of the modified smectites. Each sample was analyzed twice. For each sample, the amount of TTA^+^ (*w*_TTA^+^_EA_; wt%) corresponds to the sum of all elements except sulfur. For comparison, *w*_TTA^+^_TGA_ values are provided in the last column

	N	C	H	S	*w*_TTA^+^_EA_	*w*_TTA^+^_ TGA_
MMT-M (1)	0.97	13.86	3.17	0.03	18.00	19.8
MMT-M (2)	0.96	13.83	3.18	0.03	17.97	19.8
BEI-M (1)	0.67	9.70	2.43	0.00	12.80	14.5
BEI-M (2)	0.69	9.67	2.44	0.00	12.80	14.5
NON-M (1)	0.95	13.71	3.44	0.00	18.09	19.9
NON-M (2)	0.95	13.63	3.45	0.00	18.03	19.9

XRPD analysis of the original MMT, BEI, and NON (Fig. S2) confirmed the dominant phases of montmorillonite, beidellite, and nontronite minerals, respectively, with minor amounts of quartz (in all samples) and muscovite (in BEI and NON). A comparison of the positions of the basal reflections in the XRPD patterns of original and modified smectites (Fig. S3) shows an increase in *d*_001_ values for each of the modified smectites. The higher *d*_001_ values found for the modified smectites compared to original smectites (Table 2) indicate the intercalation of TTA^+^ into the interlayer space.

**Table 2:** Basal spacings (*d*_001_; in Å) for each original (orig.) and modified (M) smectite as obtained from the XRPD analysis (Fig. S3) are listed together with percentage change in *d*_001_ value (%*d*_001_ = 100 × *d*_001_M/*d*_001_orig.)

	*d*_001_orig.	*d*_001_M	%*d*_001_
MMT	15.17	18.51	122.0
BEI	14.95	18.05	120.7
NON	14.74	18.31	124.2

**Fig. 2:**
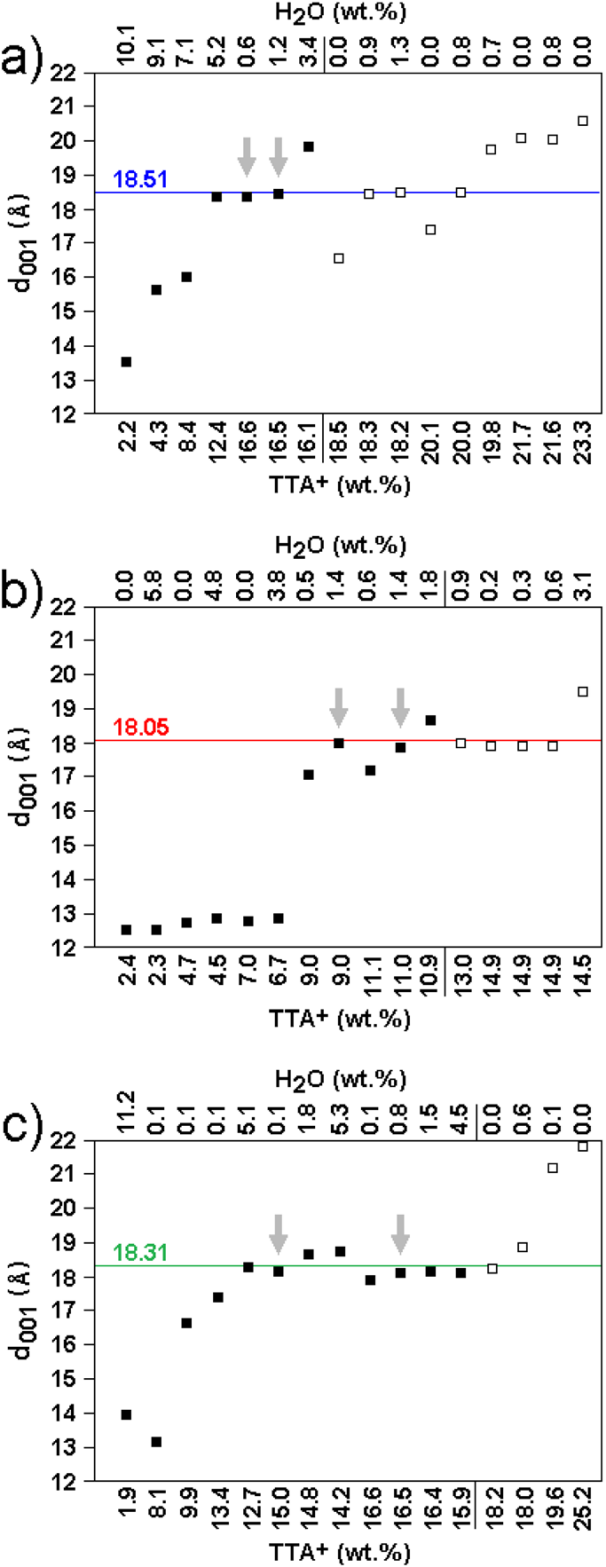
The content of TTA^+^ (wt%) and H_2_O (wt%) in the optimized models, and corresponding *d*_001_ values obtained by simulated diffraction (squares) compared to experimentally determined *d*_001_ values (horizontal lines) are shown for MMT-M (a), BEI-M (b), and NON-M (c). Models agreeing with the experimental results of elemental analysis are represented by solid squares. Models corresponding to real samples are indicated by grey arrows.

In order to determine what proportion of TTA^+^ (of its total amount in the sample) is in the interlayer space and how it is arranged there, molecular modeling was used. The *d*_001_ values obtained by simulated diffraction from optimized models of modified smectite1s with different content of TTA^+^, Na^+^, and H_2_O in the interlayer space were compared with *d*_001_ values of real samples (Fig. 2). Models containing the same or less amount of TTA^+^ than the amount determined by elemental analysis (Table 1) were selected (solid squares in Fig. 2). Among these selected models, only those with *d*_001_ values matching the experimental *d*_001_ values (colored horizontal lines in Fig. 2) and containing <1.5 wt% H_2_O can be considered as models corresponding to real samples (grey arrows in Fig. 2).

These six selected models contain 8 TTA^+^ in MMT, 4 or 5 TTA^+^ in BEI, and 8 or 9 TTA^+^ in NON (Table S3). Comparison of the *w*_TTA^+^_EA_ values (Table 1) with the *w*_TTA^+^_ values calculated from the models (Table S3) reveals that ∼90% (for MMT and NON) and ∼80% (for BEI) of TTA^+^ in the samples is located in the interlayer space.

Five of the six models show the paraffin orientation of TTA^+^ (Fig. S4a–e). The sixth one (NON with 8 TTA^+^) shows that a bilayer arrangement is also possible (Fig. S4f). These selected models were subsequently used to prepare the models of modified smectites with drugs (see Section 3.3).

### 3.2 Adsorption onto the modified smectites

**Fig. 3:**
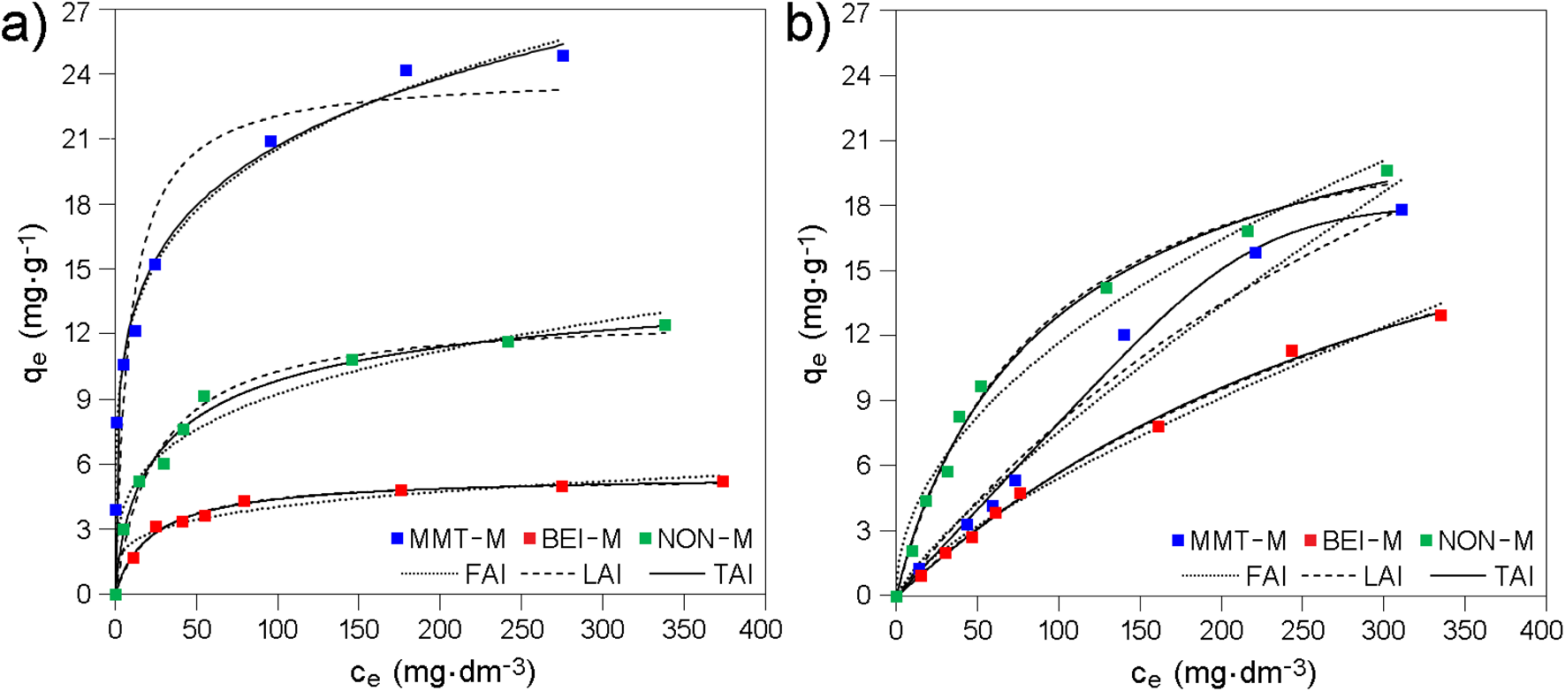
Adsorption equilibrium data, *i.e.* the dependence of equilibrium adsorption capacity *q*_e_ on equilibrium concentration *c*_e_ for (a) AMP and (b) LAM onto modified smectites.

Adsorption experiments revealed both differences between modified smectites in adsorption of the same drug and differences between drugs in adsorption on the same modified smecti1te (Fig. 3). The experimental data were fitted by FAI and LAI (Fig. 3 and Table S4). Since the LAI proved to be a more accurate model (with the exception of MMT-M/AMP), the data were also fitted with the TAI (Fig. 3 and Table S4).

The generally higher *R*^2^ values for LAI and TAI compared to FAI indicate that the adsorption of AMP and LAM can be considered as monolayer adsorption without lateral interactions of the adsorbed drugs and that the modified smectites exhibit a heterogeneous surface with preferred adsorption sites.^73,76,80^ See also the Section 3.3.

The pH value measured at the highest drug concentration, *i.e.* 400 mg dm^−3^, after 24 h of shaking the solution containing MMT-M, BEI-M, and NON-M was 6.3, 5.5, and 6.1, respectively (for AMP) and 7.1, 6.4, and 6.8, respectively (for LAM).

The descending order of modified smectites according to *q*_m_ values determined from TAI (Table S4) is NON-M > MMT-M > BEI-M for LAM and NON-M > BEI-M for AMP. MMT-M is not included in the latter case because FAI fits the experimental data more accurately than LAI and fitting using TAI was therefore not performed. However, considering the *q*_m_ values determined from LAI (Table S4) or the *q*_e_ values (Fig. 3a), the order in the case of AMP is MMT-M > NON-M > BEI-M, *i.e.* BEI-M remains in the worst position. For LAM, the orders according to *q*_m_ determined from TAI and according to *q*_e_ (Fig. 3b) are identical and also in this case the BEI-M has the worst position. It can be stated that the maximum adsorbed amount is higher for LAM compared to AMP, and that the BEI-M exhibits the lowest adsorption efficiency.

Small number of studies dealing with similar compounds allows only a limited comparison with our adsorption experiments (Table 3). Li *et al.*^2^ adsorbed AMP on MMT modified with dodecyl dimethyl benzyl ammonium, and for the same amount of adsorbent as in this study (0.1 g) and for initial AMP concentration (180 mg dm^−3^), the reported *q*_e_ (30.86 mg g^−1^) is approximately one third higher compared to *q*_e_ = 20.91 mg g^−1^ obtained in our experiments for the similar AMP concentration of 200 mg dm^−3^ (Table 3). However, considering the 4.5× higher initial amount of drug and the 2× higher amount of modifier used by Li *et al.*,^2^ the adsorption in our study can be considered more efficient. Weng *et al.*,^8^ adsorbing AMP onto nano Fe/Ni modified bentonite, used the same amount of adsorbent (0.1 g) and the same initial AMP concentration (20 mg dm^−3^) as in this study (Table 3). Due to the higher volume, the initial amount of drug was one-fifth higher (0.5 mg) compared to this study (0.4 mg), and reported *q*_e_ = 4.3 mg g^−1^ (Table 3) is comparable to 3.88 mg g^−1^ reached in our experiments (Table 3). Zusman *et al.*^53^ adsorbed LAM on MMT modified with poly-4-vinylpyridine (50% substituted with ethanol) and for an the same initial LAM concentration (20 mg dm^−3^) reported *q*_e_ = 0.64 mg g^−1^. Due to the unspecified volume, neither the initial amount of adsorbent (given in g dm^−3^) nor the initial amount of drug can be determined, however this *q*_e_ is one half lower compared to *q*_e_ = 1.26 mg g^−1^ obtained for MMT-M in our experiment (Table 3). The *q*_e_ reported by Zusman *et al.*^53^ is also one-third lower and more than three times lower than the *q*_e_ obtained under comparable conditions for BEI-M and NON-M in our experiments (Table 3). Anggraini *et al.*,^54^ adsorbing AMP on MMT pre-treated with hydrogen peroxide and modified with myristyl trimethylammonium bromide, used the same amount of adsorbent (0.1 g), similar initial AMP concentration (286.5 mg dm^−3^), and reported *q*_e_ = 49.9 mg g^−1^ (Table 3). Although this *q*_e_ value is double the 24.15 mg g^−1^ obtained in our experiments, given the nearly 6× higher initial amount of adsorbent used by Anggraini *et al.* (28.65 mg *vs.* 6.0 mg; Table 3), the experiment in our study can be considered more efficient. It can be stated that in the adsorption of AMP, the MMT-M can compete with similar adsorbents reported by other authors. In the case of LAM, all three MMT-M, BEI-M, and NON-M can compete with similar adsorbent reported by other authors.

**Table 3:** The drug adsorbed onto a given clay, either modified (mod) or original (−), the amount of modifier *w*_mod_ (wt%), the amount of adsorbent *m*_ads_ (g), the concentration of the drug *c*_drug_ (mg dm^−3^), the volume of the solution with the given drug *V*_drug_ (dm^3^), the amount of the drug in the solution *m*_drug_ (mg), equilibrium amount of drug on adsorbent *q*_e_ (mg g^−1^) and time of adsorption *t* (h). References (Ref.) to this study (TS) and to comparable works by other authors are also listed. NP – not provided^a^

Drug	Clay/mod	*w*_mod_	*m*_ads._	*c*_drug_	*V*_drug_	*m*_drug_	*q*_e_	*t*	Ref.
AMP	MMT/TTAB	∼18	0.1	200	0.02	4.0	20.91	24	TS
AMP	MMT/TTAB	∼18	0.1	20	0.02	0.4	3.88	24	TS
AMP	MMT/TTAB	∼18	0.1	300	0.02	6.0	24.15	24	TS
LAM	MMT/TTAB	∼18	0.1	20	0.02	0.4	1.26	24	TS
LAM	BEI/TTAB	∼13	0.1	20	0.02	0.4	0.94	24	TS
LAM	NON/TTAB	∼18	0.1	20	0.02	0.4	2.06	24	TS
AMP	MMT/—	0	0.1	200	0.02	4.0	21.47	24	TS
AMP	MMT/—	0	0.1	20	0.02	0.4	2.03	24	TS
AMP	MMT/—	0	0.1	300	0.02	6.0	34.58	24	TS
AMP	MMT/DBAC	∼37	0.1	180	0.12	21.6	30.86	4	2
AMP	bent./nFN	NP	0.1	20	0.025	0.5	4.25	1	8
LAM	MMT/PVP	16.5	NP*	20	NP	NP	0.64	24	53
AMP	MMT(P)/MTAB	19.85	0.1	286.5	0.10	28.65	49.9	24	54
AMP	MMT/—	0	0.1	180	0.12	21.6	0.36	4	2
AMP	MMT/—	NP	NP**	25	NP	NP	NP^#^	2	9
AMP	MMT/—	0	0.1	286.5	0.10	28.65	27.6	24	54

a AMP – ampicillin; MMT – montmorillonite; TTAB – tetradecyl trimethylammonium bromide; LAM – lamotrigine; DBAC – dodecyl dimethyl benzyl ammonium chloride; bent. – bentonite; nFN – nano Fe/Ni pillaring; PVP – poly-4-vinylpyridine (50% substituted with ethanol); NP* – *m*_ads_ provided as 1.7 g dm^−3^; MMT(P) – MMT pre-treated with hydrogen peroxide; MTAB – myristyl trimethylammonium bromide; NP** – *m*_ads_ provided as 0.5 g dm^−3^; NP^#^ – only *q*_max_ provided (141.22 mg g^−1^).

### 3.3 Structure of modified smectites after adsorption

XRPD analysis of the modified smectites before and after the adsorption showed a change in basal reflections (Table 4, Fig. S5 and Table S5). While in the case of BEI-M and NON-M the *d*_001_ values increased after the adsorption, a slight decrease was found in the case of MMT-M. However, even the slightly lower *d*_001_ values may not be inconsistent with the entry of drugs into the interlayer space, as some previous studies have shown.^66,81^ This was also confirmed by molecular modeling. For this purpose, each selected interlayer space model of each modified smectite (see Section 3.1) was supplemented with one AMP or LAM. This number is a good approximation of the drug content according to *q*_m_ (Tables S6 and S7).

**Table 4:** Basal spacings *d*_001_ (Å) for each modified smectite before (M) and after adsorption (AMP, LAM) as obtained from the XRPD analysis (Fig. S5) are listed together with percentage changes in *d*_001_ values (%*d*_001_ = 100 × *d*_001_/*d*_001_M)

	M	AMP	LAM		
	*d*_001_	*d*_001_	%*d*_001_	*d*_001_	%*d*_001_
MMT-M	18.51	17.74	95.8	17.93	96.9
BEI-M	18.05	19.20	106.4	19.72	109.3
NON-M	18.31	22.19	121.2	22.99	125.6

**Fig. 4:**
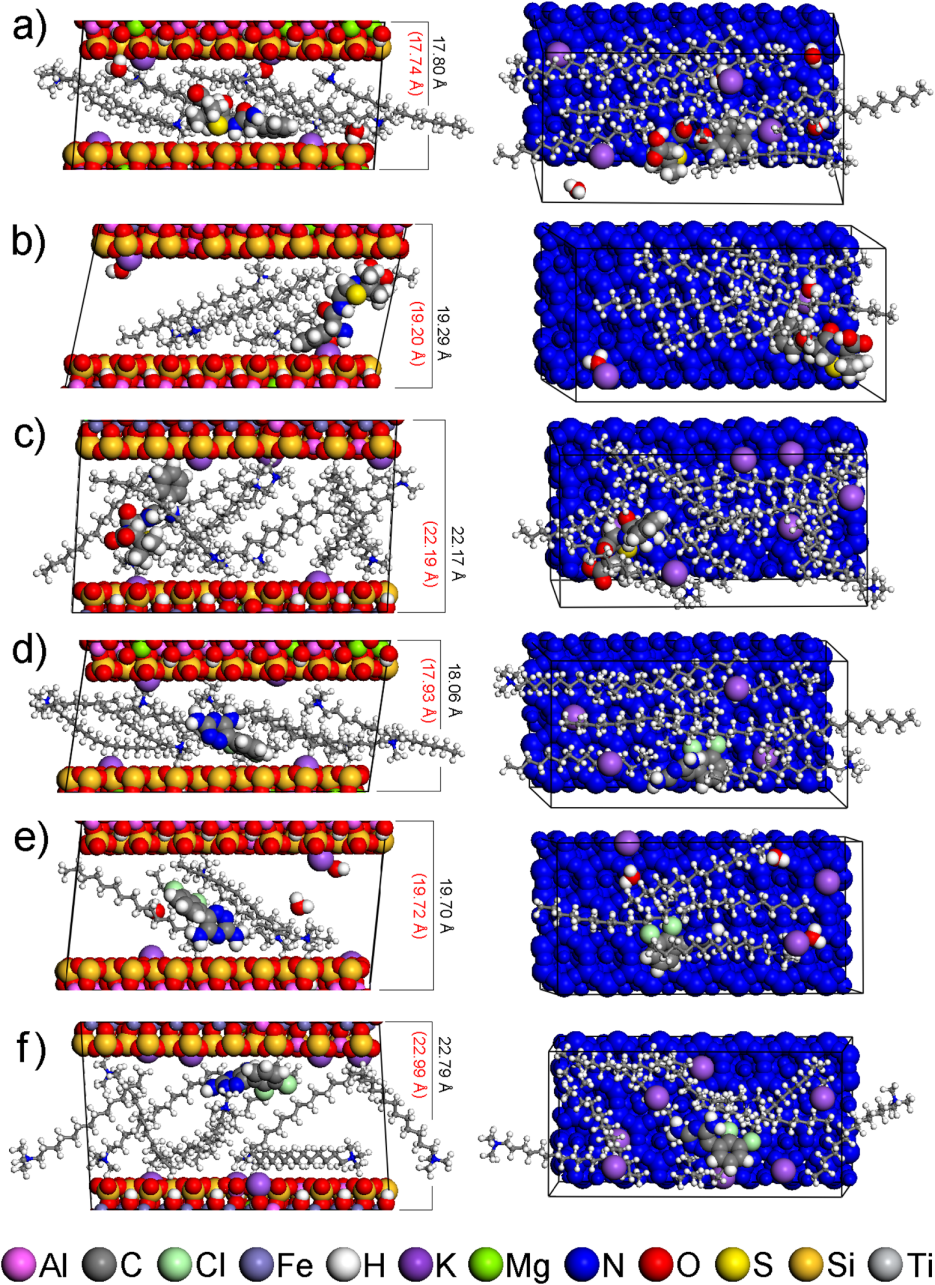
Side views (left) and top views (right) of models of the interlayer space of modified smectites with drug molecule: (a) MMT-M/AMP, (b) BEI-M/AMP, (c) NON-M/AMP, (d) MMT-M/LAM, (e) BEI-M/LAM, (f) NON-M/LAM. The *d*_001_ values of the models and the experimental *d*_001_ values are written in black and red, respectively. For clarity, all TTA^+^ are in the balls-and-stick mode, and each smectite is colored blue in the top views. Composition of the displayed models together with *d*_001_ and *E*_int_ values is available in Table S8.

The agreement of *d*_001_ values calculated from the optimized models of the interlayer space (Table S8) and the experimentally determined *d*_001_ values (Table 4) shows that all modified smectites after adsorption can contain drugs in the interlayer. The models showing *d*_001_ values closest to those experimentally determined are shown in Fig. 4.

AMP and LAM molecules are always adjacent to the smectite layer (near TTA^+^ heads or Na^+^), either in whole (Fig. 4a and f) or in part (Fig. 4b–e), with AMP in some cases adjacent with its opposite parts to both smectite layers (Fig. 4b and c). In none of the models is the interaction observed only with the nonpolar alkyl chains of TTA^+^, which is understandable considering the polarity of AMP and LAM.

**Fig. 5:**
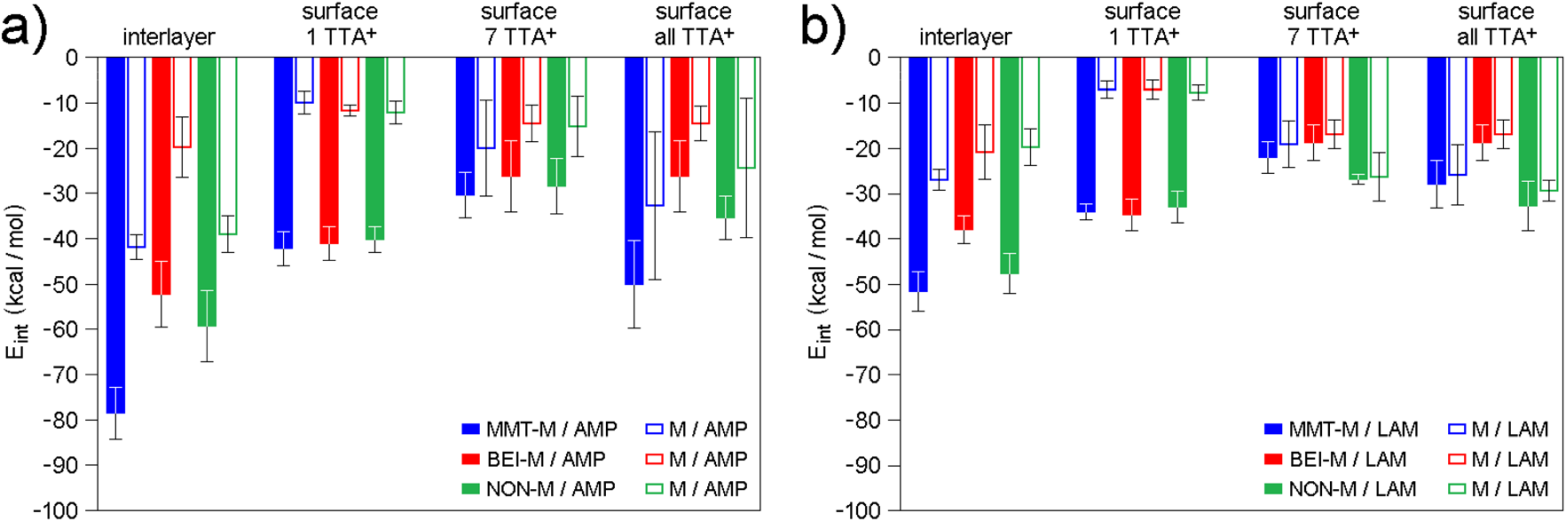
Average interaction energies (*E*_int_) of (a) AMP and (2b) LAM molecules either with the interlayer or the surface of modified smectites (filled bars) or only with TTA^+^ (empty bars) in the interlayer or the surface of modified smectites (with different numbers of TTA^+^). In the case of surface all TTA^+^, 12, 7, and 15 TTA^+^ were on the (001) surface of MMT, BEI, and NON, respectively. For more information about the models, the reader is referred to Tables S8, S9 and Fig. 4, S6–S8.

In the case 1of AMP, the descending order of smectites according to *q*_m_ values (MMT-M > NON-M > BEI-M; Table S4) is consistent with the ascending order according to *E*_int_ values calculated from optimized models of AMP in the interlayer (MMT-M < NON-M < BEI-M; see interlayer in Fig. 5a). The lowest *E*_int_ indicating the strongest modified smectite/AMP interaction was found for MMT-M, the highest *E*_int_ indicating the weakest modified smectite/AMP interaction was found for BEI-M.

Since not all TTA^+^ are in the interlayer (see Section 3.1), models representing the surface of smectite particles with different numbers of TTA^+^ on the surface were also studied, from 1 to the maximum number compensating the layer charge, *i.e.* 12, 7, and 15 TTA^+^ for MMT-M, BEI-M, and NON-M, respectively (Table S9 and Fig. S6–S8). The *E*_int_ values found for AMP on surfaces with 1 TTA^+^ are very similar (see surface 1 TTA^+^ in Fig. 5a), but with increasing number of 1 TTA^+^ the same order as in the case of interlayer begins to appear (MMT-M < NON-M < BEI-M; see surface all TTA^+^ in Fig. 5a).

In the case of LAM, the descending order of smectites according to *q*_m_ values for LAM (NON-M > MMT-M > BEI-M; Table S4) does not agree with the ascending order according to *E*_int_ values calculated from optimized models of LAM in the interlayer (MMT-M < NON-M < BEI-M; see interlayer in Fig. 5b). However, agreement is achieved for surfaces with a higher number of TTA^+^. The ascending order according to *E*_int_ values calculated for LAM on the surface with 7 and all TTA^+^ (NON-M < MMT-M < BEI-M; Fig. 5b) corresponds to the descending order of smectites according to *q*_m_ values for LAM (*i.e.*, stronger interaction agrees with higher adsorbed amount). The agreement of the modeling results for the surface with 7 and all TTA^+^ molecules with the adsorption results indicate that the preferential adsorption of LAM occurs on the surface of modified smectites rather than in the interlayer.

Since the *E*_int_ values for LAM are higher (*i.e.* the interaction is weaker) compared to the *E*_int_ values for AMP (Fig. 5), the experimentally observed better adsorption of LAM compared to AMP (in the case of NON-M and BEI-M; Fig. 3) cannot simply be a consequence of a stronger interaction between the drug and the modified smectite itself. The lower water solubility of LAM (170 mg dm^−3^)^82^ compared to the water solubility of AMP (10 100 mg dm^−3^)^83^ undoubtedly also plays a role. Only in the case of AMP on MMT does the strong interaction (see the lowest *E*_int_ in Fig. 5a) seem to contribute significantly to the observed highest adsorption efficiency of MMT (Fig. 3).

Further findings were obtained by analyzing the interaction of the drug with only TTA^+^ molecules from the given model (Fig. 5). For each model and each modified smectite, the percentage of the total *E*_int_ value attributable to the interaction of AMP or LAM with only TTA^+^ was determined (denoted as *P*_AMP/TTA^+^_ and *P*_LAM/TTA^+^_; see Table S10). In the case of the interlayer models, there is no significant difference between AMP and LAM, their interaction with TTA^+^ represents on average ∼50% of the *E*_int_ value (Table S10). For all surface models, however, the drug–TTA^+^ interaction becomes stronger with increasing amount of TTA^+^, more significantly for LAM compared to AMP. In surface 1 TTA^+^ models, there is not yet a significant difference between AMP and LAM, the interaction of a drug with a single TTA^+^ is on average ∼25% of the *E*_int_ value (Table S10). However, in models with a higher number of TTA^+^, the AMP–TTA^+^ and LAM–TTA^+^ interaction is on average ∼60% and ∼90%, respectively, of the *E*_int_ value (Table S10). These results imply that LAM on the surface of modified smectites interacts more strongly with TTA^+^ than with the smectite layer.

It is noteworthy that the order of modified smectites according to *P*_AMP/TTA^+^_ or *P*_LAM/TTA^+^_ values (Table S10) does not generally correspond to the order according to *q*_m_ values (*i.e.* NON-M > MMT-M > BEI-M). A match can be found only in the case of LAM in the surface 1 TTA^+^ models (see Table S10). Molecular modeling thus suggests that the modification of smectites does not simply lead to an enhancement of their own adsorption efficiencies, which they would exhibit in the original form, and at the same time that the adsorption efficiency of modified smectites is not controlled only by the strength of the drug–TTA^+^ interaction. The drug–smectite and drug–TTA^+^ interactions are therefore not additive, and knowledge of the adsorption efficiency of modified smectites does not provide information about the adsorption efficiency of the original smectites, which may be different. For the above reasons, further adsorption experiments were performed with the original smectites without TTA^+^.

### 3.4 Adsorption onto the original smectites

**Fig. 6:**
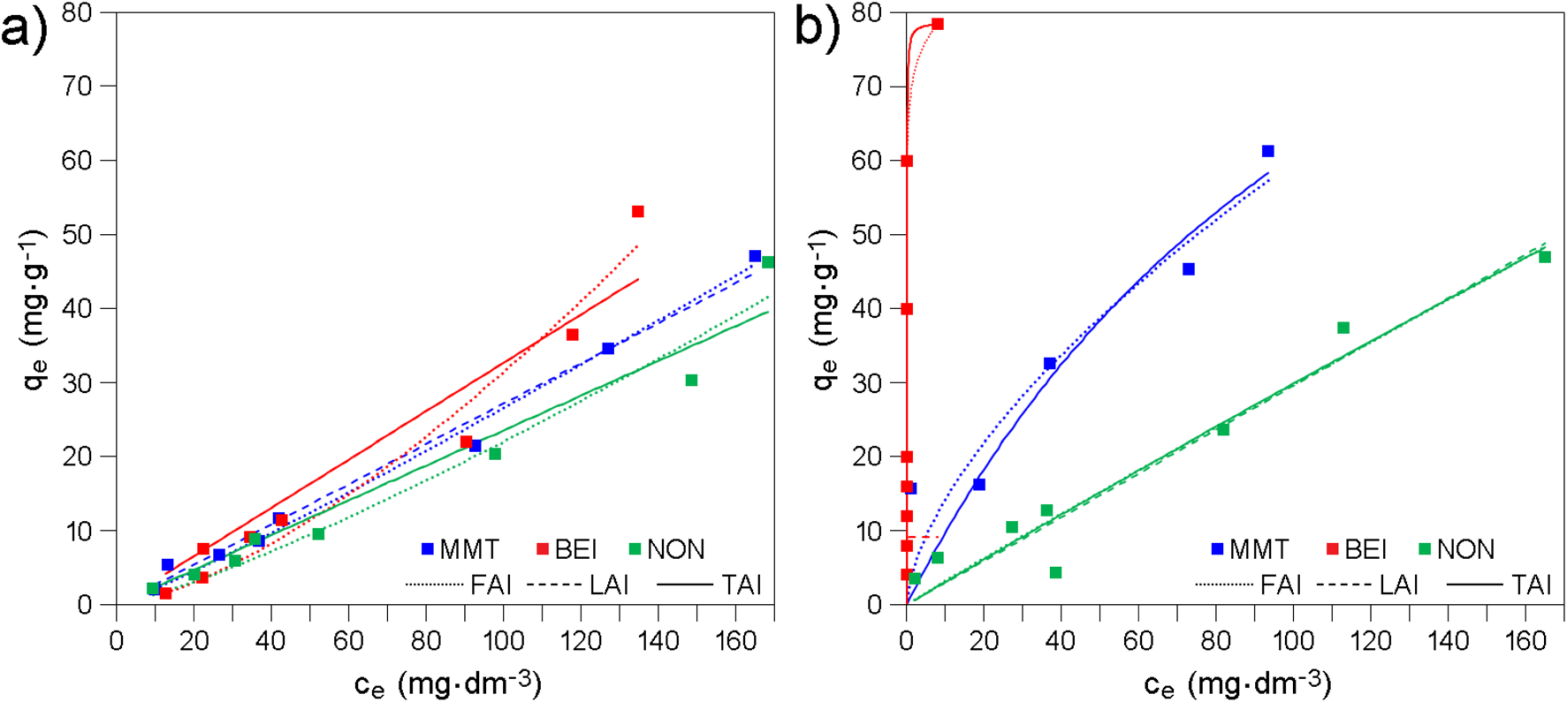
Adsorption equilibrium data, *i.e.* the dependence of equilibrium adsorption capacity *q*_e_ on equilibrium concentration *c*_e_ for (a) AMP and (b) LAM onto original smectites.

Adsorption efficiencies of the original smectites (Fig. 6) were found to be on average several times higher compared to modified smectites (Fig. 3). According to the highest *q*_e_ values determined for the original and modified smectites (compare Fig. 3 and 6; see also Table S11), the amount of AMP adsorbed on MMT, BEI, and NON is ∼2, ∼10, and ∼4 times higher, respectively, compared to the amount of AMP adsorbed on MMT-M, BEI-M, and NON-M, respectively. The amount of LAM adsorbed on MMT, BEI, and NON is ∼3, ∼6, and ∼2 times higher, respectively, compared to the amount of LAM adsorbed on MMT-M, BEI-M, and NON-M, respectively (Table S11). Also, the order of the original smectites according to the highest *q*_e_ (Fig. 6) is different (both for AMP and LAM): BEI > MMT > NON. Last but not least, the FAI best fits the experimental data (Table S12), *i.e.* the adsorption of AMP and LAM onto original smectites cannot be considered as monolayer and without lateral interactions of the drug molecules. Intermolecular interactions already come into play here (see Section 3.5). The pH values measured at the highest drug concentration, *i.e.* 400 mg dm^−3^, after 24 h of shaking the solution containing original smectites were also slightly higher compared to the solutions containing modified smectites (see Section 3.2). For MMT, BEI, and NON, the pH was 7.1, 6.3, and 7.2, respectively (for AMP) and 7.9, 6.5, and 7.5, respectively (for LAM). The only similarity in the adsorption of AMP and LAM onto original smectites and modified smectites is that the adsorbed amount of LAM is higher compared to AMP (except of MMT-M).

Studies of LAM adsorption onto original smectites are lacking in the literature. In the case of AMP, Li *et al.*^2^ adsorbed it on original MMT (Table 3). For the same amount of adsorbent as in this study (0.1 g) and for initial AMP concentration (180 mg dm^−3^), the reported *q*_e_ (0.36 mg g^−1^) is ∼60× times lower compared to *q*_e_ = 21.47 mg g^−1^ obtained in our experiments for the similar AMP concentration of 200 mg dm^−3^ (Table 3). The *q*_e_ reported by Li *et al.*^2^ is also ∼61× lower and ∼57× lower than the *q*_e_ obtained for BEI-M and NON-M in our experiments (Table 3). The six times shorter adsorption time compared to our experiment (4 h *vs.* 24 h; Table 3) could have played a role. Balarak *et al.*^9^ described the adsorption of AMP (initial concentration of 25 mg dm^−3^) onto original MMT from Iran (the amount is not provided). The *q*_e_ value is not provided, but the reported *q*_max_ = 141.22 mg g^−1^ (Fig. 6) is very high and confirms the suitability of using original MMT. Anggraini *et al.*^54^ adsorbed AMP on original MMT, and for the same amount of adsorbent (0.1 g) and similar initial AMP concentration (286.5 mg dm^−3^) reported *q*_e_ = 27.6 mg g^−1^ (Table 3). Despite almost 6× higher initial amount of adsorbent (28.65 mg *vs.* 6.0 mg; Table 3) due to the larger volume used, this *q*_e_ is a quarter lower than the 34.58 mg g^−1^ obtained for MMT-1M in our experiment (Table 3). The *q*_e_ reported by Anggraini *et al.*^54^ is also one-third lower and one-fifth lower than the *q*_e_ obtained for BEI-M and NON-M in our experiments (Table 3). It can be stated that for the adsorption of AMP, the original MMT, BEI, and NON used in this study can compete with original MMTs reported by other authors.

### 3.5 Structure of original smectites after adsorption

**Fig. 7:**
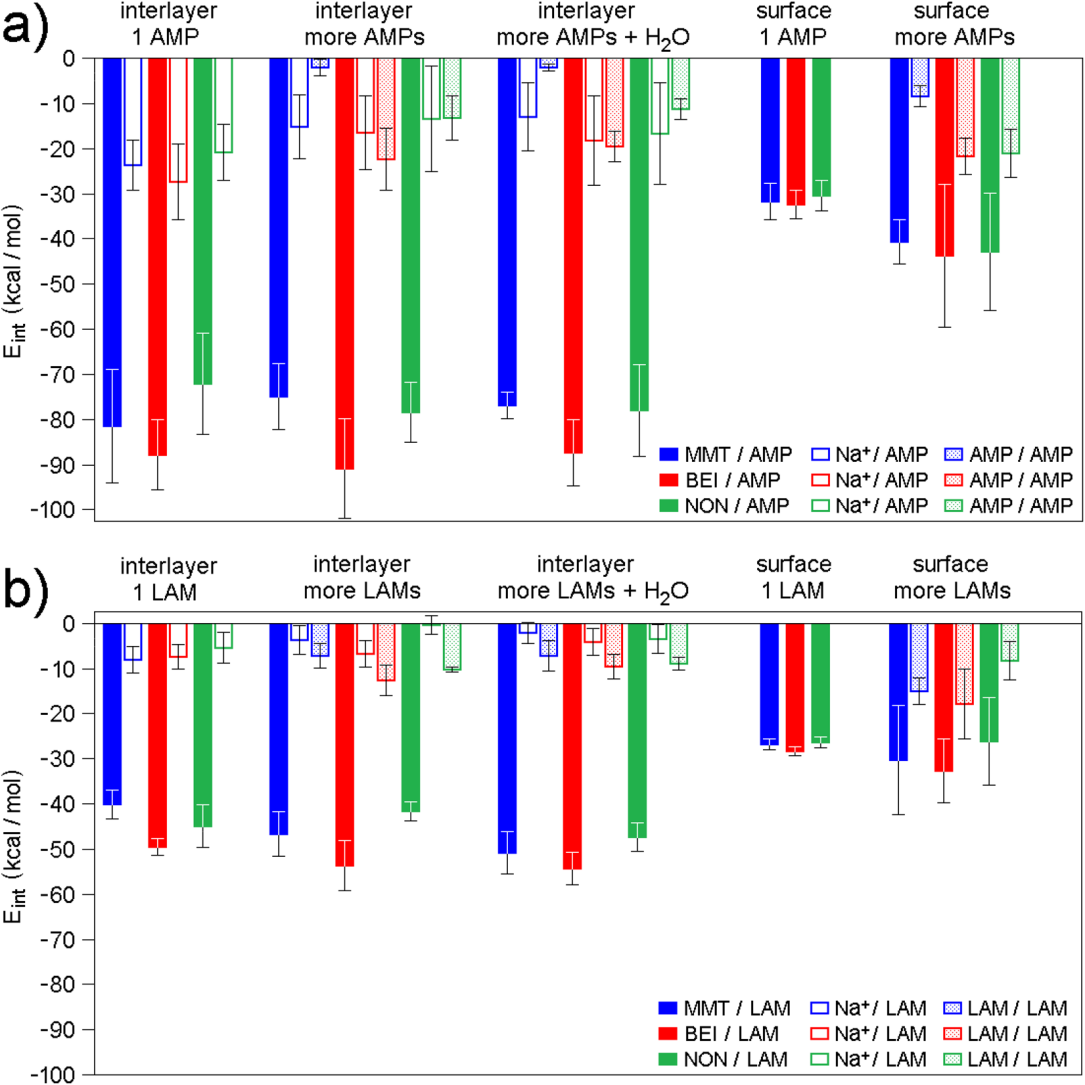
Average interaction energies (*E*_int_) of (a) AMP and (b) LAM molecules either with the interlayer of original smectites (filled bars) or only with Na^+^ cations in the interlayer (empty bars) or with the (001) surface of original smectites (filled bars). In the models containing more drug molecules (including water or not), 2, 7, 4 AMP molecules and 3, 6, 2 LAM molecules were in the interlayer of MMT, BEI, and NON, respectively. Original data and additional information are provided in Tables S14–18. Selected models are shown in Fig. 8, 9 and S10–S12.

In contrast to the modified smectites, basal reflections of the original smectites were found almost unchanged after adsorption (Table 5, Fig. S9 and Table S13). This finding, however, may not be inconsistent with the entry of drugs into the interlayer space^66,81^ as already mentioned in Section 3.3, and it is also confirmed by molecular modeling. Optimized models containing 1 drug molecule in the waterless interlayer (see interlayer 1 AMP and interlayer 1 LAM in Fig. 7a and b) exhibit good agreement of *E*_int_ values with the adsorption (Fig. 6): see the lowest *E*_int_ values, *i.e.* the strongest smectite–drug interaction, found for BEI. The same trend, although not as pronounced, can also be observed in the case of the surface models containing 1 or more drug molecules (see surface 1 AMP, surface more AMPs in Fig. 7a, and surface 1 LAM, surface more LAMs in Fig. 7b).

**Table 5:** Basal spacings *d*_001_ (Å) for each original smectite before (orig.) and after adsorption (AMP, LAM) as obtained from the XRPD analysis (Fig. S9) are listed together with percentage change in *d*_001_ value (%*d*_001_ = 100 × *d*_001_/*d*_001_M)

	Orig.	AMP	LAM		
	*d*_001_	*d*_001_	%*d*_001_	*d*_001_	%*d*_001_
MMT	15.17	15.08	99.4	15.04	99.1
BEI	14.95	14.82	99.1	14.49	96.9
NON	14.74	14.99	101.7	14.91	101.2

**Fig. 8:**
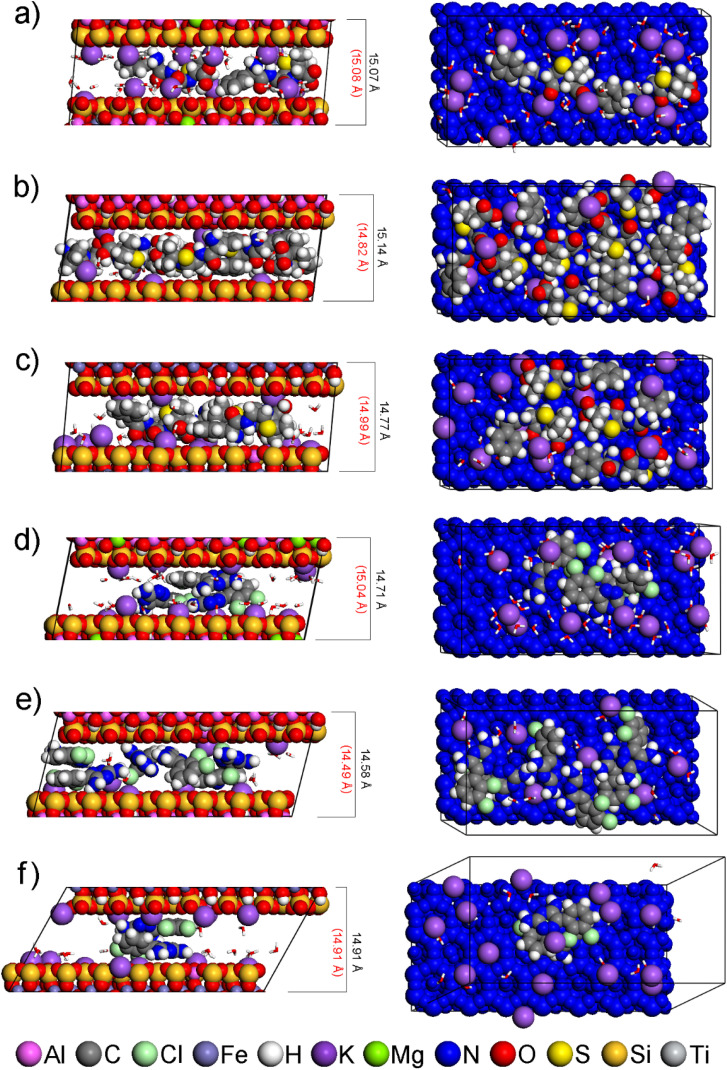
Side views (left) and top views (right) of models of the interlayer space of original smectites with more drug molecules (*i.e.* 2, 7, 4 AMPs and 3, 6, 2 LAMs for MMT, BEI, NON, respectively) and with water: (a) MMT/AMP, (b) BEI/AMP, (c) NON/AMP, (d) MMT/LAM, (e) BEI/LAM, (f) NON/LAM. The *d*_001_ values of the models and the experimental *d*_001_ values are written in black and red, respectively. For clarity, each smectite is colored blue in the top views and all water molecules are displayed in stick mode. Composition of the displayed models together with *d*_001_ and *E*_int_ values is available in Table S16.

However, the models interlayer 1 AMP and interlayer 1 LAM cannot be considered as corresponding to reality, since their computed *d*_001_ values are too low (Table S14) compared to *d*_001_ values of real samples (Table 5 and Fig. S10). Only an increase in the number of drug molecules (in accordance with the increased adsorbed amount of drugs, as described in Section 3.4) in the waterless interlayer space led to the similarity of computed *d*_001_ values (Table S15) with *d*_001_ values of real samples (Table 5 and Fig. S11). The agreement of *E*_int_ trends (see interlayer more AMPs in Fig. 7a and interlayer more LAMs in Fig. 7b) with the experiment was maintained. Finally, the addition of water molecules to these models resulted in agreement with both adsorption, in terms of *E*_int_ trends (see interlayer more AMPs + H_2_O in Fig. 7a and interlayer more LAMs + H_2_O in Fig. 7b), and with XRPD analysis, in terms of comparable computed and experimental *d*_001_ values (Table 5 and Table S16; see also Fig. 8).

In addition to the total interactions, partial interactions of drug molecules with only Na^+^ cations or with only other drug molecules were also analyzed (Fig. 7). These analyses revealed differences between AMP and LAM. In the AMP/MMT models, the AMP–Na^+^ interaction is significantly stronger compared to the AMP–AMP interaction (Fig. 7a). A similar situation occurs in the case of AMP/NON models, where, considering the standard deviation of *E*_int_ values, the AMP–Na^+^ interaction can also be considered stronger compared to the AMP–AMP (Fig. 7a). In the case of AMP/BEI, the AMP–AMP interaction slightly exceeds the AMP–Na^+^ interaction, but only in models with no water (Fig. 7a). In contrast, the LAM–LAM interaction is stronger compared to the LAM–Na^+^ interaction in all smectites with no exception (Fig. 7b).

These results can be interpreted as AMP being preferentially adsorbed *via* interaction with Na^+^ and the adsorption of additional AMP molecules is no longer as strong. The exception observed in the case of interlayer more AMPs models for BEI/AMP (Fig. 7a) agrees well with the highest *q*_e_ value for AMP on BEI (Fig. 6). On the other hand, the preferential adsorption of LAM through strong interactions with other LAM, rather than through weaker interactions with Na^+^, allows for the adsorption of more and more LAM molecules onto smectite. These stronger LAM–LAM interactions (Fig. 7b) suggest one of the causes of the higher *q*_e_ for LAM compared to AMP (Fig. 6).

**Fig. 9:**
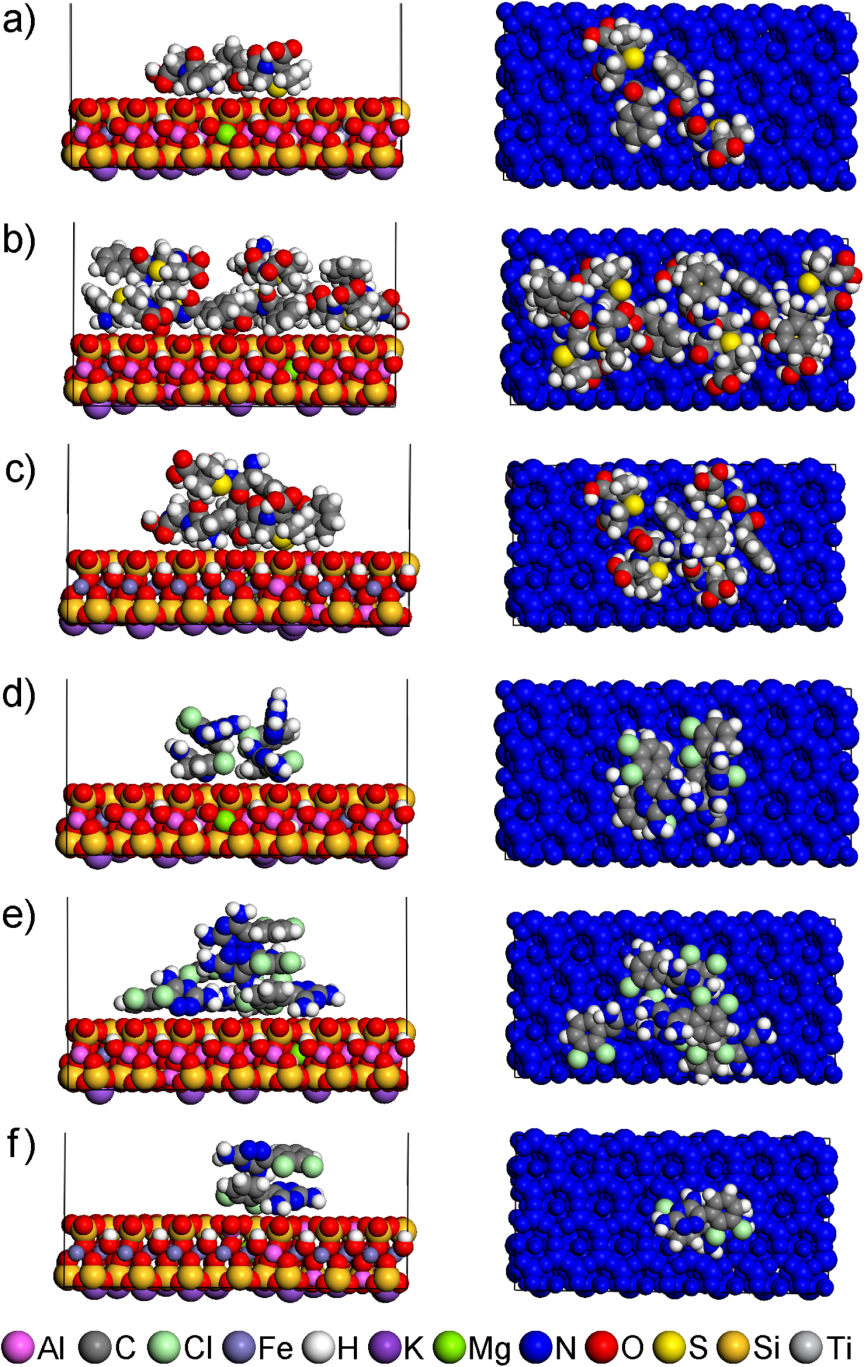
Side views (left) and top views (right) of the models of the surfaces of original smectites with more drug molecules (*i.e.* 2, 7, 4 AMPs and 3, 6, 2 LAMs for MMT, BEI, NON, respectively): (a) MMT/AMP, (b) BEI/AMP, (c) NON/AMP, (d) MMT/LAM, (e) BEI/LAM, (f) NON/LAM. For clarity, each smectite is colored blue in the top views. Composition of the displayed models together with *E*_int_ values is available in Table S18.

The LAM molecules tend to stack on top of each other both in the interlayer and on the surface (Fig. S11d–f, 8d–f and 9d–f). AMP molecules exhibit this beh1avior only on the surface and only when there are three or more of them (Fig. 9a–c). This can be demonstrated by the different behavior of two AMP (on the MMT surface; Fig. 9a) and two LAM (on the NON surface; Fig. 9f). Geometry optimization of two AMP placed one on top of the other always results in the position of the AMP next to each other, both touching the smectite surface (Fig. 9a). In contrast, two LAM placed one on top of the other always retain this position after geometry optimization (Fig. 9f) due to the advantageous flat shape and the attraction of aromatic rings.

The mere comparison shows overall lower *E*_int_ values for models with AMP (Fig. 7a) compared to models with LAM (Fig. 7b), which in itself contradicts the results of adsorption experiments. However, as in the Section 3.3, it is important to note that the experimentally observed higher adsorption of LAM compared to AMP (Fig. 6) is not simply a consequence of a stronger smectite–drug interaction. The models describe the situation when the drug has already reached the smectite and do not include the behavior of the drug in the surrounding aqueous environment. And as in the case of modified smectites, the difference in solubility of AMP and LAM in water^82,83^ plays an important role. Only the combination of the energetically advantageous stacking of LAM molecules and the significantly lower solubility of LAM in water leads to an explanation of the experimentally observed higher adsorption of LAM.

### 3.6 Reproducibility of the adsorption onto the original smectites

**Fig. 10:**
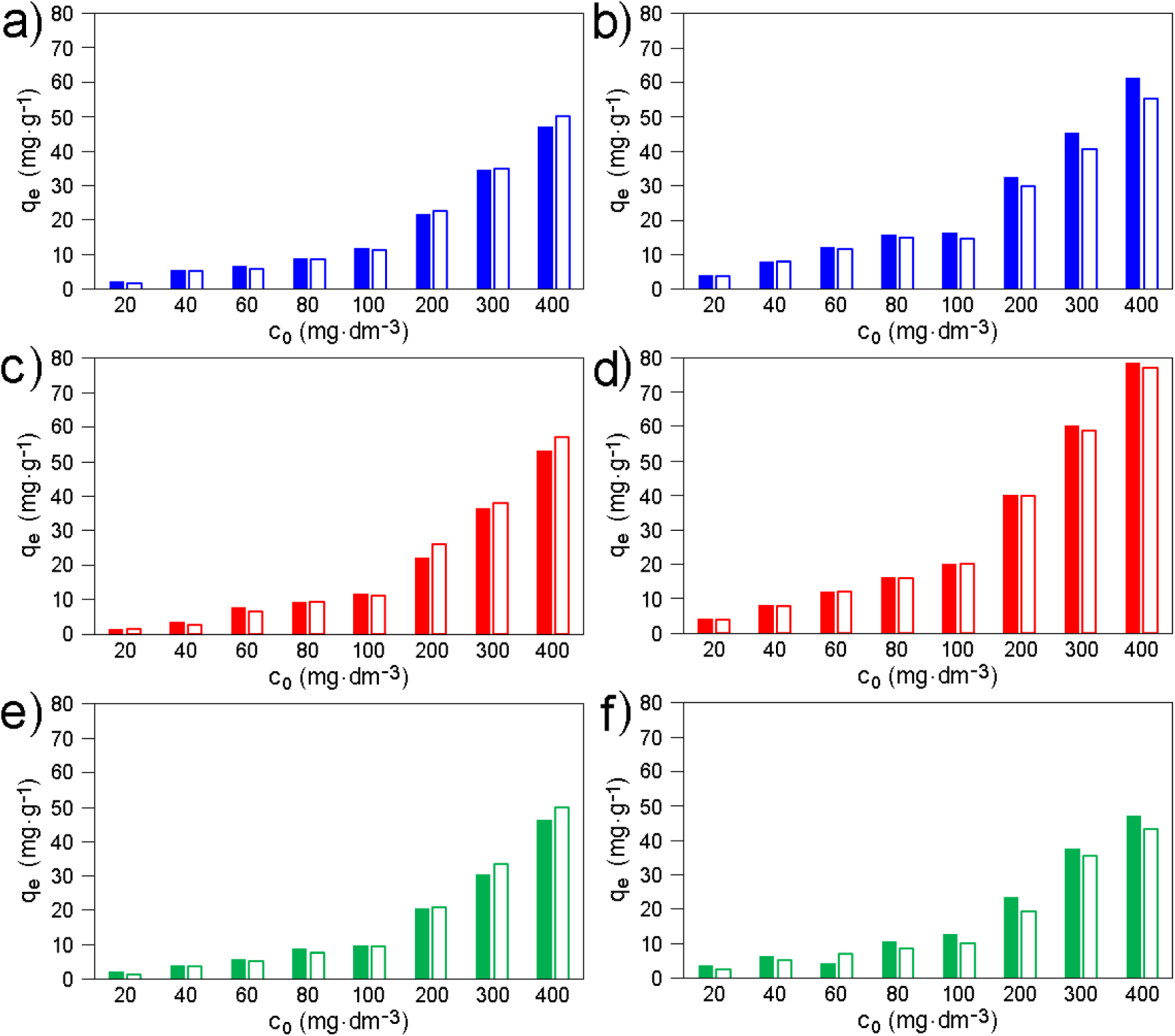
Comparison of *q*_e_ values from the first (filled bars) and the second (empty bars) adsorption experiment for (a) MMT/AMP, (b) MMT/LAM, (c) BEI/AMP, (d) BEI/LAM, (e) NON/AMP, and (f) NON/LAM in dependence on *c*_0_ values. The numerical values of *q*_e_, *c*_0_, and *c*_e_ are provided in Table S19.

Repeated adsorption experiments demonstrate good reproducibility of drug adsorption onto the original smectites (Fig. 10 and Table S19). The *r*(*q*_e_) values, calculated using eqn (7), for AMP on MMT, BEI, and NON are 104.57%, 101.70%, and 106.13%, respectively, for LAM on MMT, BEI, and NON are 106.24%, 100.49%, and 110.92%, respectively (see also Table S19).

In the case of AMP, the differences between the *q*_e_ values from the first and second (repeated) adsorption are larger at lower concentrations, *i.e.* for *c*_0_ in the range of 20–80 mg dm^−3^ (Table S19). This dependence was not observed for LAM, which can be explained by the significantly higher water solubility of LAM compared to AMP.^82,83^ An important result is that the order of the original smectites according to the highest *q*_e_ values, *i.e.*, BEI > MMT > NON, is preserved for both AMP and LAM in the repeated adsorption experiments (Fig. 10 and Table S19).

## 4 Conclusions

Smectites modified with TTA^+^ (MMT-M, BEI-M, NON-M) adsorb AMP and LAM drugs from water in amounts ranging from units to lower tens of mg per 1 g of adsorbent. Since the Toth and Langmuir models fit the adsorption data better than the Freundlich model (except for AMP on MMT-M), the adsorption can be considered monolayer. The following order of modified smectites according to adsorption efficiency was found: MMT-M > NON-M > BEI-M and NON-M > MMT-M > BEI-M for AMP and LAM, respectively. It correlates with the layer charges and consequently with the amount of TTA^+^ in the modified smectites. The higher adsorbed amount of LAM compared to AMP is related to the lower solubility of LAM in water. Molecular modeling showed that the adsorption efficiency of modified smectites is not controlled only by the strength of the drug–TTA^+^ interaction.

The amount of drugs adsorbed onto original smectites (MMT, BEI, NON) is higher compared to modified smectites and reaches or exceeds (for LAM on BEI and NON) 50 mg per 1 g of adsorbent. The order of original smectites according to adsorption efficiency is BEI > MMT > NON for both AMP and LAM. The amount of adsorbed LAM is again higher compared to AMP. Since the Freundlich model fits the adsorption data better than the Toth and Langmuir models, the adsorption can no longer be considered as a monolayer. Molecular modeling showed that while AMP is preferentially adsorbed through interaction with Na^+^ and the interaction with other AMP molecules is weaker, LAM is preferentially adsorbed through interactions with other LAM molecules, rather than through weaker interactions with Na^+^. This finding (together with the low water solubility of LAM) explains the higher adsorbed amount of LAM.

Repeated adsorption experiments revealed good reproducibility of AMP and LAM adsorption onto the original MMT, BEI, and NON. Since the original smectites are cheap and widely available, their regeneration and recycling were not addressed in this study. However, one possibility for further use of the original smectites after adsorption (containing ∼7 wt% adsorbate or less – see the highest *q*_e_ value of ∼78.4 mg LAM per 1 g BEI) could be, *e.g.*, ceramics, during firing of which the organics would be destroyed.

The results of our study can be briefly summarized as follows. (1) Modification did not enhance the adsorption efficiency of original smectites. (2) Original smectites showed higher adsorption efficiency compared to the modified ones. (3) Original smectites are a suitable environmentally friendly alternative to the commonly used QACs-modified smectites.

Further research is needed and the authors encourage other researchers to include original smectites in their experiments with QACs-modified smectites. There are still very few studies that provide such comparisons. However, only a sufficient amount of data will lead to an answer to the following question: is it really necessary to purify waters containing harmful substances using adsorbents based on smectites modified with QACs, which also pose an environmental risk? Our study suggests that the answer could be: no.

## Author contributions

Jonáš Tokarský: data curation, formal analysis, funding acquisition, investigation, methodology, project administration, supervision, visualization, writing – original Draft, writing – review & editing Pavlína Peikertová: formal analysis, investigation, writing – original draft, writing – review & editing Klára Výšková: data curation, formal analysis, investigation Markéta Davidová: investigation Silvie Vallová: data curation, conceptualization, investigation, methodology, resources, supervision, validation, writing – review & editing.

## Conflicts of interest

There are no conflicts to declare.

## Supplementary Material

RA-015-D5RA04769B-s001

## Data Availability

Original data are available. See DOI: https://doi.org/10.5281/zenodo.15574791. Supplementary information is available. See DOI: https://doi.org/10.1039/d5ra04769b.
