# Supplementary material for: Natural versus organophilized smectites as drug adsorbents: experiment and molecular modeling
Source: RSC Adv. 2025 Sep 30;15(43):36065–83. doi: 10.1039/d5ra04769b (PMC12481201; doi:10.1039/d5ra04769b)
Supplement: RA-015-D5RA04769B-s001 [file RA-015-D5RA04769B-s001.pdf]

Supplementary Information (SI) for RSC Advances.
This journal is © The Royal Society of Chemistry 2025

Supplementary material for

Natural versus organophilized smectites as drug adsorbents: experiment and molecular modeling

Jonáš Tokarský, Pavlína Peikertová, Klára Výšková, Markéta Davidová, Silvie Vallová

Table S1

Composition (in. wt.%) of original smectites MMT, BEI, and NON determined by X-ray fluorescence spectroscopy analysis.

	MMT	BEI	NON
Al_2O_3	18.78	27.63	10.49
CaO	0.31	0.21	0.13
Fe_2O_3	1.12	2.09	33.54
K_2O	0.06	0.16	0.28
P_2O_5	0.007	0.002	–
MgO	4.26	0.45	0.19
MnO	0.003	–	–
Na_2O	1.85	0.84	0.95
SiO_2	70.53	65.58	53.07
TiO_2	0.36	0.95	0.0002
ZnO	0.0001	–	–

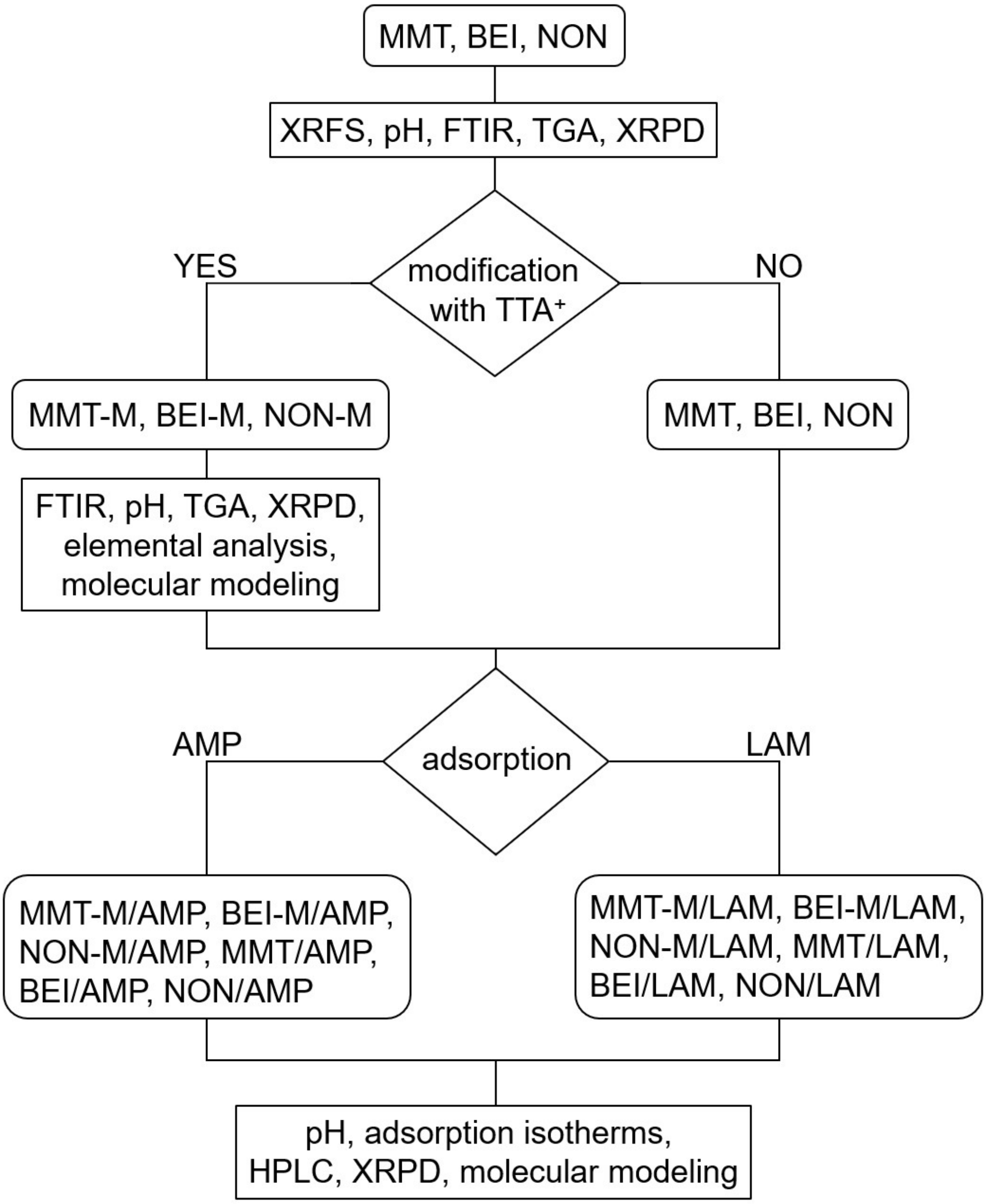

Fig. S1 The processing of smectites and the subsequent sequence of experiments and analyses performed in this study.

Crystallochemical formulas of smectites

MMT

unit cell: $(Al_{2.97}Fe^{3+}_{0.12}Mg_{0.88}Ti_{0.03})$ (Si_8) O_{20} $(OH)_4$
total layer charge: -0.85; octahedral charge: -0.85; tetrahedral charge: 0.00

7*a*×2*b*×1*c* supercell: $(Al_{41.58}Fe^{3+}_{1.68}Mg_{12.32}Ti_{0.42})$ (Si_{112}) O_{280} $(OH)_{56}$
total layer charge: -11.90; octahedral charge: -11.90; tetrahedral charge: 0.00

7*a*×2*b*×1*c* supercell rounded: $(Al_{42}Fe^{3+}_{2}Mg_{12})$ (Si_{112}) O_{280} $(OH)_{56}$
total layer charge: -12.00; octahedral charge: -12.00; tetrahedral charge: 0.00

BEI

unit cell: $(Al_{3.61}Fe^{3+}_{0.21}Mg_{0.09}Ti_{0.09})$ $(Si_{7.53}Al_{0.47})$ O_{20} $(OH)_4$
total layer charge: -0.47; octahedral charge: 0.00; tetrahedral charge: -0.47

7*a*×2*b*×1*c* supercell: $(Al_{50.54}Fe^{3+}_{2.94}Mg_{1.26}Ti_{1.26})$ $(Si_{105.42}Al_{6.58})$ O_{280} $(OH)_{56}$
total layer charge: -6.58; octahedral charge: 0.00; tetrahedral charge: -6.58

7*a*×2*b*×1*c* supercell rounded: $(Al_{51}Fe^{3+}_{3}Mg_{1}Ti_{1})$ $(Si_{105}Al_{7})$ O_{280} $(OH)_{56}$
total layer charge: -7.00; octahedral charge: 0.00; tetrahedral charge: -7.00

NON

unit cell formula: $(Al_{0.59}Fe^{3+}_{3.36}Mg_{0.05})$ $(Si_{6.98}Al_{1.02})$ O_{20} $(OH)_4$
total layer charge: -1.07; octahedral charge: -0.05; tetrahedral charge: -1.02

7*a*×2*b*×1*c* supercell: $(Al_{8.26}Fe^{3+}_{47.04}Mg_{0.70})$ $(Si_{97.72}Al_{14.28})$ O_{280} $(OH)_{56}$
total layer charge: -14.98; octahedral charge: -0.70; tetrahedral charge: -14.28

7*a*×2*b*×1*c* supercell rounded: $(Al_{8}Fe^{3+}_{47}Mg_{1})$ $(Si_{98}Al_{14})$ O_{280} $(OH)_{56}$
total layer charge: -15.00; octahedral charge: -1.00; tetrahedral charge: -14.00

Calculations of interaction energies

The drug–TTA^+ interaction energy $E_{int,D\text{-}TTA}$ (kcal mol^{-1}) was calculated using the eqn (3a)

$$E_{int,D\text{-}TTA} = E_{tot} - (E_D + E_{TTA}) \quad (3a)$$

where E_{tot} (kcal mol^{-1}) is a total potential energy of the drug and all TTA^+ molecules in the optimized model from which other components were removed, E_D (kcal mol^{-1}) is a total potential energy of a drug molecule (AMP or LAM), and E_{TTA} (kcal mol^{-1}) is a total potential energy of all TTA^+ molecules.

The drug–Na^+ interaction energy $E_{int,D\text{-}Na}$ (kcal mol^{-1}) was calculated using the eqn (3b)

$$E_{int,D\text{-}Na} = E_{tot} - (E_D + E_{Na}) \quad (3b)$$

where E_{tot} (kcal mol^{-1}) is a total potential energy of the drug and all Na^+ cations in the optimized model from which other components were removed, E_D (kcal mol^{-1}) is a total potential energy of a drug molecule (AMP or LAM), and E_{Na} (kcal mol^{-1}) is a total potential energy of all Na^+ cations.

The drug–drug interaction energy $E_{int,D(i)\text{-}D}$ (kcal mol^{-1}) was calculated using the eqn (3c)

$$E_{int,D(i)\text{-}D} = E_{tot} - (E_{D(i)} + E_D) \quad (3c)$$

where E_{tot} (kcal mol^{-1}) is a total potential energy of all drug molecules in the optimized model from which other components were removed, $E_{D(i)}$ (kcal mol^{-1}) is a total potential energy of the i-th drug molecule (AMP or LAM), and E_D (kcal mol^{-1}) is a total potential energy of all drug molecules without the i-th drug molecule.

In the case of pure smectite models containing multiple drug molecules, the interaction energy of each i-th drug molecule with smectite $E_{int,D(i)\text{-}sm}$ (kcal mol^{-1}) was calculated using the eqn (3d)

$$E_{int,D(i)\text{-}sm} = E_{tot} - (E_{D(i)} + E_{w/D}) \quad (3d)$$

where E_{tot} (kcal mol^{-1}) is a total potential energy of the whole optimized model from which all drug molecules except the i-th drug molecule were removed, $E_{D(i)}$ (kcal mol^{-1}) is a total potential energy of the i-th drug molecule (AMP or LAM), and $E_{w/D}$ (kcal mol^{-1}) is a total potential energy of the optimized model from which all drug molecules including the i-th drug molecule were removed.

Similarly for the drug–Na^+ interaction in the case of pure smectite models containing multiple drug molecules, the interaction energy of each i-th drug molecule the cations $E_{int,D(i)\text{-}Na}$ (kcal mol^{-1}) was calculated using the eqn (3e)

$$E_{int,D(i)\text{-}Na} = E_{tot} - (E_{D(i)} + E_{Na}) \quad (3e)$$

where E_{tot} (kcal mol^{-1}) is a total potential energy of the i-th drug and all Na^+ cations in the optimized model from which other components were removed, $E_{D(i)}$ (kcal mol^{-1}) is a total potential energy of the i-th drug molecule (AMP or LAM), and E_{Na} (kcal mol^{-1}) is a total potential energy of all Na^+ cations.

Table S2

Wavenumbers (in cm^{-1}) and assignments of significant bands in FTIR spectra of TTAB, MMT, BEI, and NON.

sample	wavenumber	assignment	Ref.
TTAB	2921	$-CH_2$ symmetric stretching	[1,2]
TTAB	2852	$-CH_2$ asymmetric stretching	[1,2]
TTAB	1470	C-H symmetric/asymmetric stretching	[1,2]
TTAB	964	$C-N^+$ stretching	[1,2]
TTAB	913	C-H *trans* out-of-plane bending	[1,2]
TTAB	720	C-H *cis* out-of-plane bending	[1,2]
MMT	3630	Al-O-H vibration	[3]
MMT	3430; 1638	H-O-H vibration	[3]
MMT	1118	Si-O vibration	[3]
MMT	1042	Si-O-Si vibration	[3]
MMT	912	Al-O-H vibration	[3]
MMT	885; 794	(Al,Mg)-O-H vibration	[3]
MMT	627	Al-O-H vibration	[3]
MMT	521	Si-O-Al vibration	[3]
MMT	461	Si-O-Mg vibration	[3]
BEI	3698	Al-OH-Al vibration	[4]
BEI	3652	Al-OH-Fe vibration	[4]
BEI	3618	Al-OH-Mg vibration	[4]
BEI	3423; 1644	H-O-H vibration	[4]
BEI	1114; 1006	Si-O vibration	[5]
BEI	1029	Si-O-Si vibration	[5]
BEI	914	Al-O-H vibration	[5]
BEI	535	Al-O-Si vibration	[5]
BEI	469	Si-O vibration	[5]
NON	3696; 3619; 3570; 819	Fe-O-H vibration	[6]
NON	3394; 1642	H-O-H vibration	[6]
NON	1108; 1010	Si-O vibration	[6]
NON	1031	Si-O-Si vibration	[6]
NON	909	Al-Fe-OH vibration	[6]
NON	679	Mg-Fe-OH vibration	[6]
NON	537; 429	Si-O-Fe vibration	[6]
NON	469	Si-O-Si vibration	[6]

References in Table S2

[1] R. Kumar Banjare, M. Kumar Banjare and S. Panda, Effect of acetonitrile on the colloidal behavior of conventional cationic surfactants: A combined conductivity, surface tension, fluorescence and FTIR study, *J. Solution Chem.*, 2020, **49**, 34-51. http://doi.org/10.1007/s10953-019-00937-4

[2] G. Socrates, Infrared and Raman characteristic group frequencies: tables and charts. John Wiley & Sons, 2004. ISBN: 978-0-470-09307-8

[3] H. V. D. Marel and H. Beutelspacher, Atlas of infrared spectroscopy of clay minerals and their admixtures, Elsevier Science Ltd, 1976. ISBN 978-0-444-41187-7

[4] B. B. Zviagina, D. K. McCarty, J. Środoń and V. A. Drits, Interpretation of infrared spectra of dioctahedral smectites in the region of OH-stretching vibrations, *Clays Clay Miner.*, 2004, **52(4)**, 399-410. https://doi.org/10.1346/CCMN.2004.0520401

[5] J. Kyziol-Komosinska, F. Barba, P. Callejas and C. Rosik-Dulewska, Beidellite and other natural low-cost sorbents to remove chromium and cadmium from water and wastewater, *Bol. Soc. Esp. Ceram. Vidr.*, 2010, **49**, 121-128.

[6] R. L. Frost, J. T. Kloprogge and Z. Ding, The Garfield and Uley nontronites—an infrared spectroscopic comparison, *Spectrochim. Acta A*, 2002, **58(9)**, 1881-1894. https://doi.org/10.1016/S1386-1425(01)00638-2

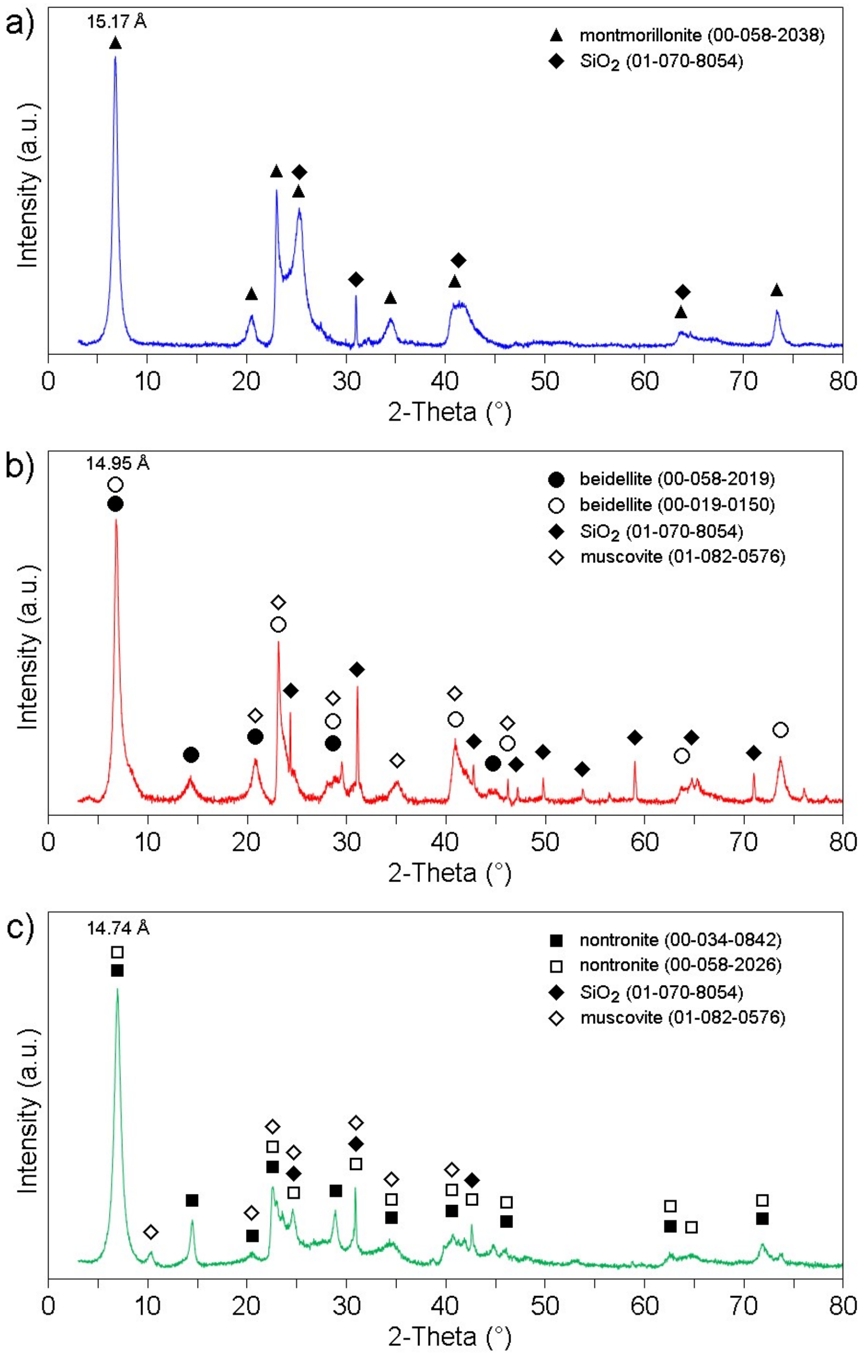

Fig. S2 XRPD patterns of original a) montmorillonite, b) beidellite, and c) nontronite with identified phases including PDF numbers. Basal distance for each pure smectite is also provided.

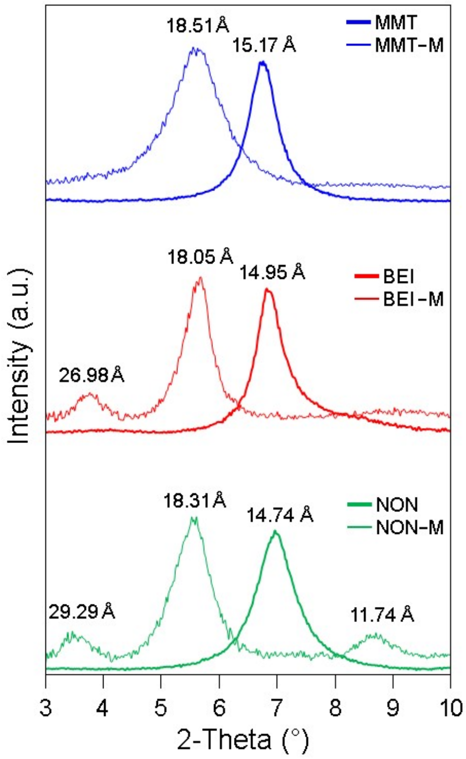

Fig. S3 Positions of basal reflections in XRPD patterns of original (MMT, BEI, NON) and modified (MMT-M, BEI-M, NON-M) smectites.

Table S3

Interlayer content in models corresponding to the real samples of modified smectites, i.e. MMT-M, BEI-M, and NON-M. The numbers (N) of TTA^+, Na^+ and H_2O in models and the calculated mass fractions (w) of TTA^+, Na^+, H_2O are listed together with the calculated mass fractions of nitrogen, carbon, and hydrogen. References to the figures of each model are provided in the last column.

smectite	N_{TTA+}	N_{Na+}	N_{H2O}	w_{TTA+}	w_N	w_C	w_H	w_{Na+}	w_{H2O}	Fig. S4
MMT	8	4	4	16.61	0.91	13.24	2.47	0.75	0.58	a
MMT	8	4	8	16.52	0.90	13.16	2.45	0.74	1.16	b
BEI	4	3	9	8.95	0.49	7.13	1.33	0.60	1.42	c
BEI	5	2	9	10.96	0.60	8.74	1.63	0.39	1.39	d
NON	8	7	1	15.00	0.82	11.95	2.23	1.18	0.13	e
NON	9	6	6	16.48	0.90	13.14	2.45	0.99	0.77	f

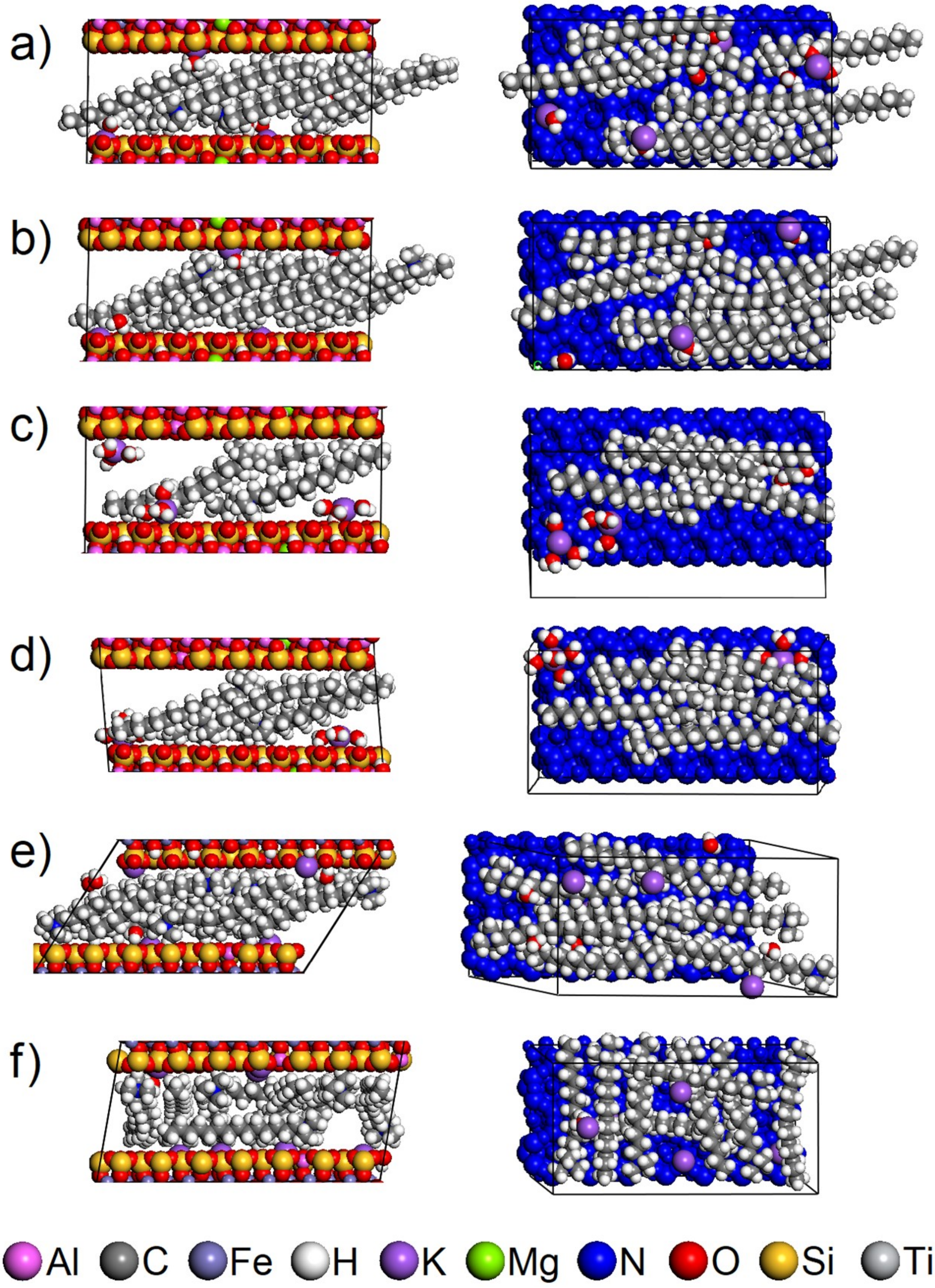

Fig. S4 Side views (left) and top views (right) of the models corresponding to real samples of the modified smectites (see Table S2). (a) MMT with 16.61 wt.% of TTA^+. (b) MMT with 16.52 wt.% of TTA^+. (c) BEI with 8.95 wt.% of TTA^+. (d) BEI with 10.96 wt.% of TTA^+. (e) NON with 15.00 wt.% of TTA^+. (f) NON with 16.48 wt.% of TTA^+. For better clarity, each smectite is colored blue in the top views. Composition of the displayed models is available in Table S3.

Table S4

Freundlich, Langmuir and Toth adsorption isotherm parameters for the adsorption of each drug (AMP, LAM) onto each modified smectite (MMT-M, BEI-M, NON-M) are listed. In addition to maximum adsorption capacities (q_m) and constants of adsorption isotherms (K_F, K_L, K_T, n), correlation coefficients (R^2) quantifying the match of a given isotherm with experimental data are also provided.

Freundlich	q_m	K_F	n	R^2
MMT-M / AMP	–	7.612	4.634	0.995
BEI-M / AMP	–	1.386	4.313	0.959
NON-M / AMP	–	2.534	3.562	0.971
MMT-M / LAM	–	0.174	1.220	0.973
BEI-M / LAM	–	0.172	1.333	0.993
NON-M / LAM	–	1.202	2.026	0.979
**Langmuir**	q_m	K_L	n	R^2
MMT-M / AMP	23.990	0.114	–	0.899
BEI-M / AMP	5.421	0.044	–	0.992
NON-M / AMP	12.939	0.039	–	0.983
MMT-M / LAM	32.155	0.002	–	0.980
BEI-M / LAM	29.822	0.002	–	0.997
NON-M / LAM	23.373	0.012	–	0.993
**Toth**	q_m	K_T	n	R^2
MMT-M / AMP	2768.195	$4.300 \cdot 10^{10}$	0.047	0.990
BEI-M / AMP	5.570	0.050	0.893	0.993
NON-M / AMP	16.192	0.081	0.551	0.991
MMT-M / LAM	19.203	0.004	2.623	0.995
BEI-M / LAM	18.490	0.003	0.908	0.997
NON-M / LAM	20.242	0.012	0.859	0.993

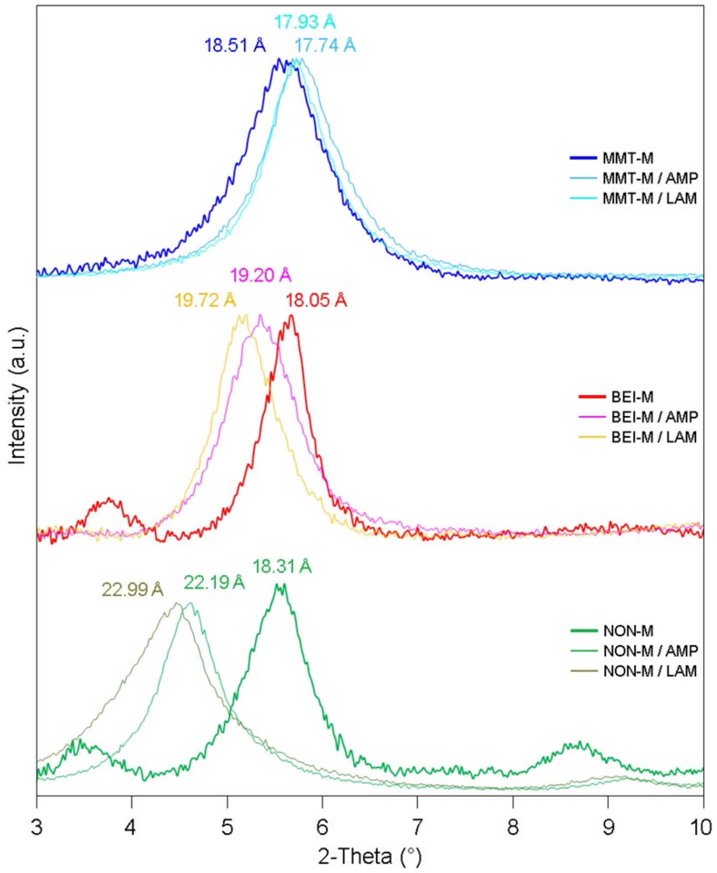

Fig. S5 Positions of basal reflections in XRPD patterns of modified smectites before adsorption (MMT-M, BEI-M, NON-M) and after adsorption of AMP (MMT-M/AMP, BEI-M/AMP, NON-M/AMP) and LAM (MMT-M/LAM, BEI-M/LAM, NON-M/LAM). Additional information is available in Table S5.

Table S5

For each modified smectite before and after the adsorption of AMP or LAM, the position of the basal reflection maximum (2θ; in °2θ), the corresponding d_{001} value (d_{001}; in Å), the full width at half maximum of the basal reflection (FWHM; in °2θ) and the corresponding intervals of positions (2θ range; in °2θ) and d_{001} values (d_{001} range; in Å) are listed.

sample	2θ	d_{001}	FWHM	2θ range	d_{001} range
MMT-M	5.54	18.51	1.02	5.08–6.10	20.18–16.81
MMT-M / AMP	5.78	17.74	0.9	5.32–6.22	19.27–16.49
MMT-M / LAM	5.72	17.93	0.78	5.35–6.13	19.17–16.73
BEI-M	5.68	18.05	0.52	5.35–5.87	19.17–17.47
BEI-M / AMP	5.34	19.20	0.85	4.93–5.78	20.80–17.74
BEI-M / LAM	5.20	19.72	0.74	4.83–5.57	21.23–18.41
NON-M	5.60	18.31	0.72	5.16–5.88	19.87–17.44
NON-M / AMP	4.62	22.19	0.76	4.22–4.98	24.29–20.59
NON-M / LAM	4.46	22.99	1.1	3.82–4.92	26.84–20.84

Table S6

The maximum adsorbed amount of AMP or LAM (q_m) determined from TAI (see Table S4), and the corresponding mass fraction of AMP or LAM. The q_m value marked with asterisk was determined from LAI (see Table S4).

AMP	q_m (mg/g)	w_{AMP} (wt.%)
MMT-M	23.990*	2.34
BEI-M	55.570	0.55
NON-M	16.192	1.59
LAM	q_m (mg/g)	w_{AMP} (wt.%)
MMT-M	19.203	1.88
BEI-M	18.490	1.82
NON-M	20.242	1.98

Table S7

The numbers (N) of TTA^+, Na^+ and H_2O in the interlayer space models corresponding to the real samples of modified smectites (see Table S2), and such a theoretical number of drug molecules (N_{AMP} or N_{LAM}) that corresponds to a mass fraction of the drug identical to the mass fraction according to the q_m (see Table S6). For each combination of N_{TTA+}, N_{Na+}, N_{H2O} in a given interlayer space model, an anhydrous variant, i.e. $N_{H2O} = 0$, is also provided. It can be seen that the values of N_{AMP} and N_{LAM} are not significantly affected by changing N_{H2O}. It is also evident that the values of N_{AMP} and N_{LAM} are quite close to 1 (with the exception of N_{AMP} in BEI), with the N_{LAM} values being slightly closer than N_{AMP} values. Therefore, 1 drug molecule was always added to each model (for comparison purposes also in the case of AMP in BEI).

smectite	N_{TTA+}	N_{Na+}	N_{H2O}	N_{AMP}	w_{AMP}
MMT	8	4	8	0.85	2.34
MMT	8	4	0	0.84	2.34
BEI	4	3	9	0.18	0.55
BEI	4	3	0	0.18	0.55
BEI	5	2	9	0.19	0.55
BEI	5	2	0	0.19	0.55
NON	8	7	1	0.63	1.59
NON	8	7	0	0.63	1.59
NON	9	6	6	0.65	1.59
NON	9	6	0	0.64	1.59

smectite	N_{TTA+}	N_{Na+}	N_{H2O}	N_{LAM}	w_{LAM}
MMT	8	4	8	0.93	1.88
MMT	8	4	0	0.91	1.88
BEI	4	3	9	0.83	1.82
BEI	4	3	0	0.81	1.82
BEI	5	2	9	0.84	1.82
BEI	5	2	0	0.83	1.82
NON	8	7	1	1.07	1.98
NON	8	7	0	1.07	1.98
NON	9	6	6	1.10	1.98
NON	9	6	0	1.09	1.98

Table S8

The numbers (N) of TTA^+, Na^+, H_2O, AMP and LAM molecules in models of the interlayer space of the modified smectites. Corresponding d_{001} (Å) and E_{int} (kcal mol^{-1}; eqn (3)) values are also listed. The E_{int} values for the drug-TTA^+ interaction ($E_{int,D\text{-}TTA}$; kcal mol^{-1}; eqn (3a)) are provided in parentheses. Models having d_{001} values closest to the experimental d_{001} values are displayed in Fig. 4 as indicated in the last column. All d_{001} values belong to the d_{001} range determined from FWHM of the basal reflection of the corresponding real sample (see Table S5).

interlayer	N_{TTA+}	N_{Na+}	N_{H2O}	N_{AMP}	N_{LAM}	d_{001}	E_{int} ($E_{int,D\text{-}TTA}$)	Fig.
MMT	8	4	4	1	0	17.80	-82.76 (-38.00)	4a
MMT	8	4	0	1	0	17.85	-85.32 (-43.15)	
MMT	8	4	4	1	0	17.93	-80.33 (-45.31)	
MMT	8	4	4	1	0	18.08	-71.31 (-38.68)	
MMT	8	4	4	1	0	18.09	-72.36 (-43.12)	
BEI	5	2	0	1	0	19.04	-46.44 (-15.20)	
BEI	5	2	2	1	0	19.29	-55.61 (-17.03)	4b
BEI	5	2	0	1	0	19.37	-65.17 (-33.21)	
BEI	5	2	0	1	0	19.49	-47.51 (-17.27)	
BEI	5	2	0	1	0	19.52	-46.42 (-16.76)	
NON	9	6	6	1	0	21.07	-65.04 (-40.47)	
NON	8	7	0	1	0	21.30	-70.45 (-43.57)	
NON	9	6	0	1	0	21.55	-49.77 (-33.06)	
NON	8	7	0	1	0	22.17	-59.31 (-42.77)	4c
NON	8	7	0	1	0	22.84	-51.07 (-35.62)	
MMT	8	4	4	0	1	17.47	-54.04 (-29.34)	
MMT	8	4	4	0	1	17.85	-48.47 (-27.35)	
MMT	8	4	0	0	1	18.06	-47.38 (-27.55)	4d
MMT	8	4	4	0	1	18.19	-48.52 (-28.70)	
MMT	8	4	4	0	1	18.43	-58.92 (-22.63)	
BEI	5	2	2	0	1	19.58	-33.99 (-17.36)	
BEI	4	3	3	0	1	19.70	-34.94 (-23.30)	4e
BEI	5	2	2	0	1	19.88	-39.91 (-17.26)	
BEI	5	2	2	0	1	19.96	-37.79 (-14.93)	
BEI	4	3	3	0	1	20.10	-42.42 (-31.27)	
NON	8	7	0	0	1	21.81	-49.85 (-19.45)	
NON	9	6	0	0	1	22.49	-53.23 (-24.16)	
NON	8	7	0	0	1	22.72	-41.73 (-14.26)	
NON	8	7	0	0	1	22.79	-42.66 (-16.89)	4f
NON	9	6	3	0	1	23.52	-50.00 (-24.67)	

Table S9

The numbers (N) of TTA^+, Na^+, AMP and LAM molecules in models of the surface of the modified smectites are listed together with total E_{int} (kcal mol^{-1}; eqn (3)) values. The E_{int} values for the drug-TTA^+ interaction ($E_{int,D-TTA}$; kcal mol^{-1}; eqn (3a)) are provided in parentheses. Models with the lowest E_{int} are displayed in Figs. S6-S8 as indicated in the last column.

surface	N_{TTA+}	N_{Na+}	N_{AMP}	N_{LAM}	E_{int} ($E_{int,D-TTA}$)	Fig.
MMT	1	11	1	0	-47.65 (-8.56)	S6a
MMT	1	11	1	0	-44.19 (-10.01)	
MMT	1	11	1	0	-41.27 (-13.44)	
MMT	1	11	1	0	-40.23 (-6.46)	
MMT	1	11	1	0	-36.64 (-10.84)	
BEI	1	6	1	0	-44.92 (-10.06)	S6b
BEI	1	6	1	0	-44.38 (-13.64)	
BEI	1	6	1	0	-41.46 (-12.52)	
BEI	1	6	1	0	-38.75 (-10.90)	
BEI	1	6	1	0	-34.96 (-11.67)	
NON	1	14	1	0	-44.14 (-13.78)	S6c
NON	1	14	1	0	-41.77 (-11.01)	
NON	1	14	1	0	-40.50 (-16.51)	
NON	1	14	1	0	-38.80 (-9.33)	
NON	1	14	1	0	-35.62 (-10.78)	
MMT	1	11	0	1	-35.19 (-7.62)	S6d
MMT	1	11	0	1	-35.18 (-8.51)	
MMT	1	11	0	1	-34.13 (-8.72)	
MMT	1	11	0	1	-33.58 (-6.71)	
MMT	1	11	0	1	-30.87 (-3.95)	
BEI	1	6	0	1	-37.38 (-8.11)	S6e
BEI	1	6	0	1	-36.11 (-8.42)	
BEI	1	6	0	1	-36.02 (-8.75)	
BEI	1	6	0	1	-35.95 (-6.38)	
BEI	1	6	0	1	-27.41 (-3.46)	
NON	1	14	0	1	-34.91 (-9.05)	S6f
NON	1	14	0	1	-34.71 (-8.34)	
NON	1	14	0	1	-34.39 (-8.29)	
NON	1	14	0	1	-34.20 (-8.77)	
NON	1	14	0	1	-25.92 (-4.56)	
MMT	7	5	1	0	-37.34 (-33.68)	S7a
MMT	7	5	1	0	-31.94 (-25.32)	
MMT	7	5	1	0	-30.20 (-23.31)	
MMT	7	5	1	0	-29.93 (-11.18)	
MMT	7	5	1	0	-21.69 (-3.19)	
BEI	7	0	1	0	-36.31 (-9.06)	S7b
BEI	7	0	1	0	-30.47 (-16.33)	
BEI	7	0	1	0	-29.70 (-16.06)	

BEI	7	0	1	0	-20.08 (-20.08)	
BEI	7	0	1	0	-14.15 (-10.95)	
NON	7	8	1	0	-39.23 (-21.79)	S7c
NON	7	8	1	0	-29.91 (-26.35)	
NON	7	8	1	0	-24.28 (-17.95)	
NON	7	8	1	0	-23.97 (-12.39)	
NON	7	8	1	0	-23.37 (-7.36)	
MMT	7	5	0	1	-26.85 (-23.19)	S7d
MMT	7	5	0	1	-25.01 (-19.50)	
MMT	7	5	0	1	-21.61 (-7.34)	
MMT	7	5	0	1	-19.63 (-17.27)	
MMT	7	5	0	1	-16.58 (-16.58)	
BEI	7	0	0	1	-22.80 (-15.71)	S7e
BEI	7	0	0	1	-20.48 (-20.48)	
BEI	7	0	0	1	-19.73 (-19.73)	
BEI	7	0	0	1	-18.65 (-18.01)	
BEI	7	0	0	1	-10.99 (-10.99)	
NON	7	8	0	1	-28.28 (-28.28)	S7f
NON	7	8	0	1	-27.78 (-11.86)	
NON	7	8	0	1	-26.43 (-25.38)	
NON	7	8	0	1	-26.10 (-23.66)	
NON	7	8	0	1	-24.96 (-24.63)	
MMT	12	0	1	0	-65.18 (-38.55)	S8a
MMT	12	0	1	0	-55.14 (-55.14)	
MMT	12	0	1	0	-48.44 (-9.55)	
MMT	12	0	1	0	-44.50 (-38.18)	
MMT	12	0	1	0	-36.63 (-19.90)	
BEI	7	0	1	0	-36.31 (-9.06)	S8b
BEI	7	0	1	0	-30.47 (-16.33)	
BEI	7	0	1	0	-29.70 (-16.06)	
BEI	7	0	1	0	-20.08 (-20.08)	
BEI	7	0	1	0	-14.15 (-10.95)	
NON	15	0	1	0	-39.36 (-38.42)	S8c
NON	15	0	1	0	-37.74 (-6.67)	
NON	15	0	1	0	-36.73 (-36.73)	
NON	15	0	1	0	-36.21 (-36.21)	
NON	15	0	1	0	-25.83 (-4.23)	
MMT	12	0	0	1	-32.41 (-32.41)	S8d
MMT	12	0	0	1	-31.45 (-31.45)	
MMT	12	0	0	1	-31.40 (-31.40)	
MMT	12	0	0	1	-25.13 (-16.51)	
MMT	12	0	0	1	-18.38 (-18.38)	
BEI	7	0	0	1	-22.80 (-15.71)	S8e
BEI	7	0	0	1	-20.48 (-20.48)	
BEI	7	0	0	1	-19.73 (-19.73)	
BEI	7	0	0	1	-18.65 (-18.01)	
BEI	7	0	0	1	-10.99 (-10.99)	

NON	15	0	0	1	-43.08 (-32.79)	S8f
NON	15	0	0	1	-32.10 (-26.57)	
NON	15	0	0	1	-30.57 (-30.57)	
NON	15	0	0	1	-29.21 (-29.21)	
NON	15	0	0	1	-27.65 (-27.65)	

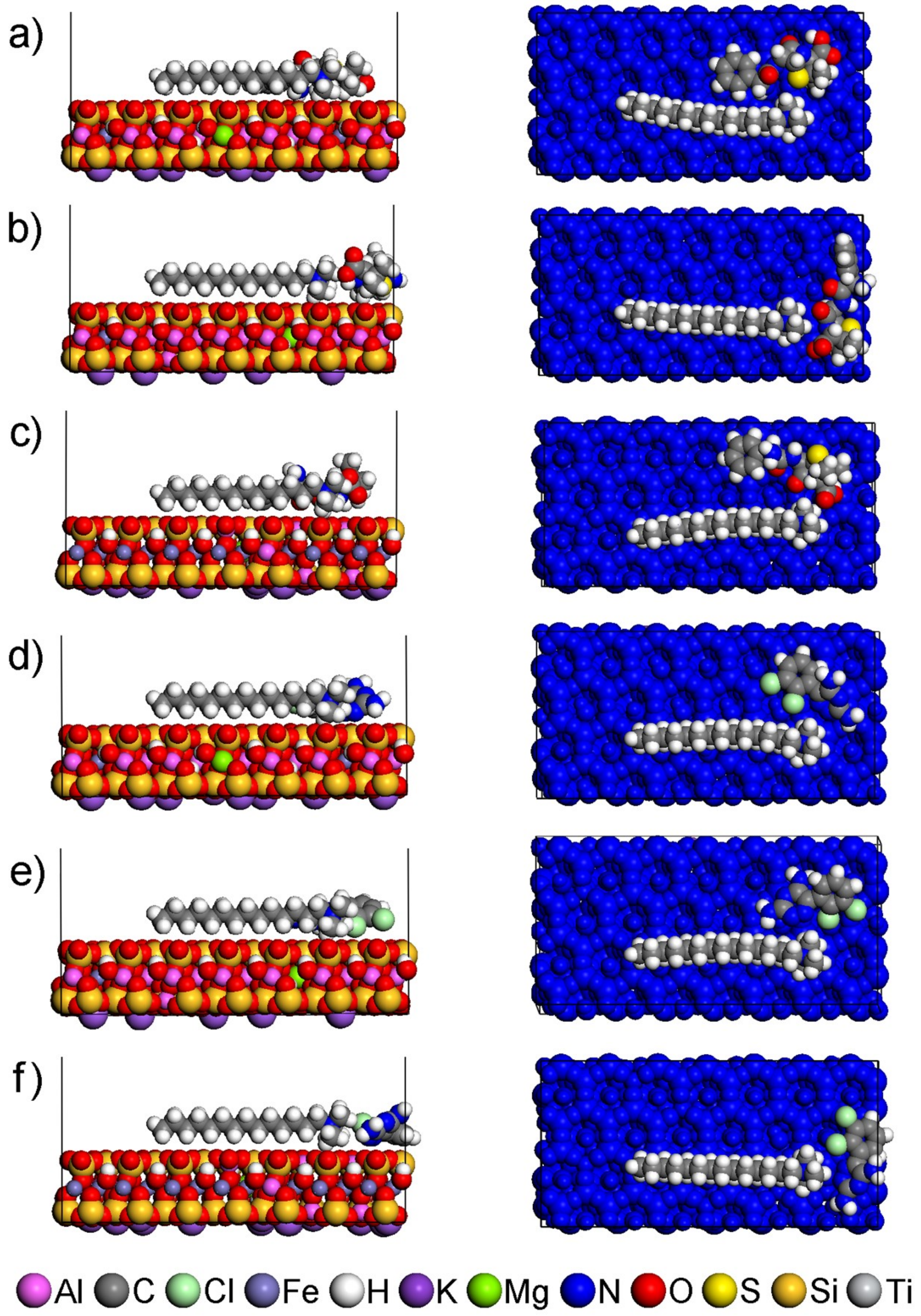

Fig. S6 Side views (left) and top views (right) of the models of the surfaces of modified smectites with 1 TTA^+ and 1 drug molecule: (a) MMT-M / AMP, (b) BEI-M / AMP, (c) NON-M / AMP, (d) MMT-M / LAM, (e) BEI-M / LAM, (f) NON-M / LAM. For better clarity, each smectite is colored blue in the top views. Composition of the displayed models together with E_{int} values is available in Table S9.

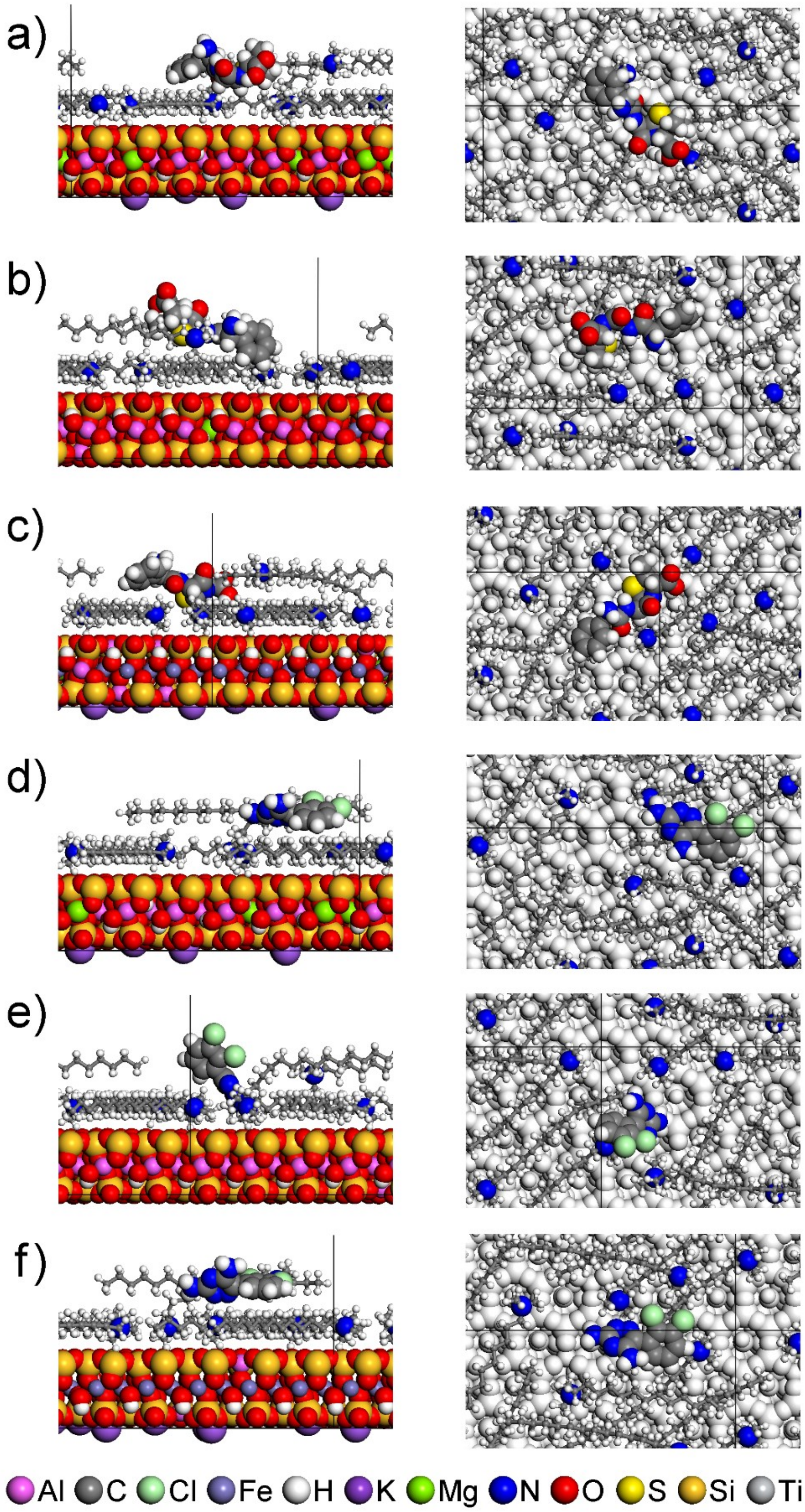

Fig. S7 Side views (left) and top views (right) of the models of the surfaces of modified smectites with 7 TTA^+ and 1 drug molecule: (a) MMT-M / AMP, (b) BEI-M / AMP, (c) NON-M / AMP, (d) MMT-M / LAM, (e) BEI-M / LAM, (f) NON-M / LAM. For better clarity, all TTA^+ are in the are in the balls-and-stick mode, and each smectite is colored white in the top views. Composition of the displayed models together with E_{int} values is available in Table S9.

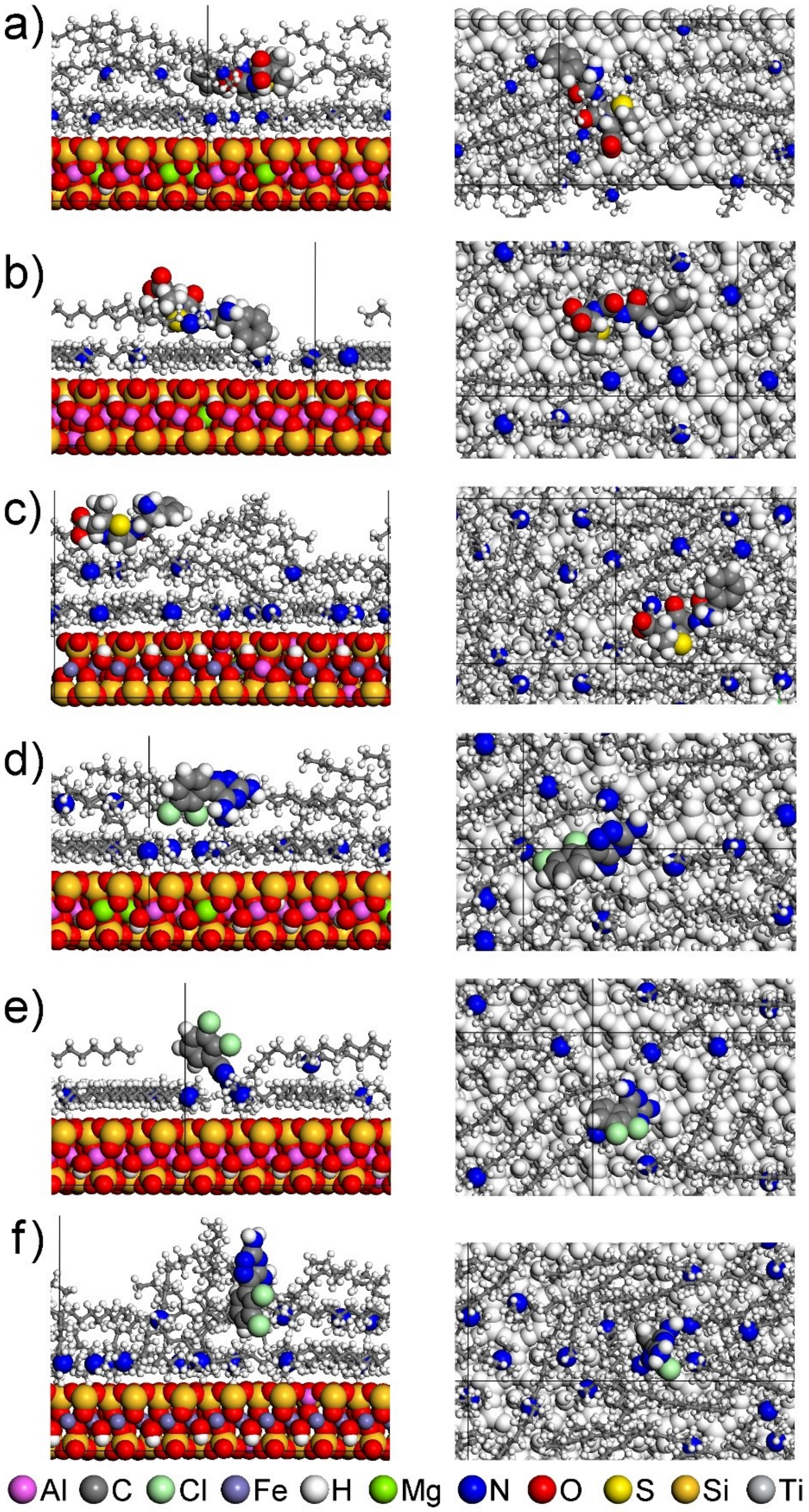

Fig. S8 Side views (left) and top views (right) of the models of the surfaces of modified smectites with all TTA^+ (i.e. 12, 7, 15 for MMT-M, BEI-M, NON-M, respectively) and 1 drug molecule: (a) MMT-M / AMP, (b) BEI-M / AMP, (c) NON-M / AMP, (d) MMT-M / LAM, (e) BEI-M / LAM, (f) NON-M / LAM. For better clarity, all TTA^+ are in the balls-and-stick mode, and each smectite is colored white in the top views. Composition of the displayed models together with E_{int} values is available in Table S9.

Table S10

For each model and each modified smectite, the percentage of the total E_{int} value attributable to the interaction of AMP or LAM with TTA^+ ($P_{AMP/TTA+}$, $P_{LAM/TTA+}$; in %) is provided. The percentage was calculated from E_{int} average values (E_{int}(avg)) and $E_{int(D\text{-}TTA)}$ average values ($E_{int(D\text{-}TTA)}$(avg)) as (100·$E_{int(D\text{-}TTA)}$(avg)) / E_{int}(avg). All numerical values of E_{int} and $E_{int(D\text{-}TTA)}$ are listed in both Table S8 and Table S9.

model	smectite	$P_{AMP/TTA+}$	$P_{LAM/TTA+}$
interlayer	MMT-M	53.1	52.7
interlayer	BEI-M	38.1	55.1
interlayer	NON-M	66.1	41.9
surface 1 TTA$^+$	MMT-M	23.5	21.0
surface 1 TTA$^+$	BEI-M	28.7	20.3
surface 1 TTA$^+$	NON-M	30.6	23.8
surface 7 TTA$^+$	MMT-M	66.4	87.2
surface 7 TTA$^+$	BEI-M	55.4	91.6
surface 7 TTA$^+$	NON-M	54.6	99.0
surface all TTA$^+$	MMT-M	64.6	93.8
surface all TTA$^+$	BEI-M	55.4	91.6
surface all TTA$^+$	NON-M	69.5	90.3

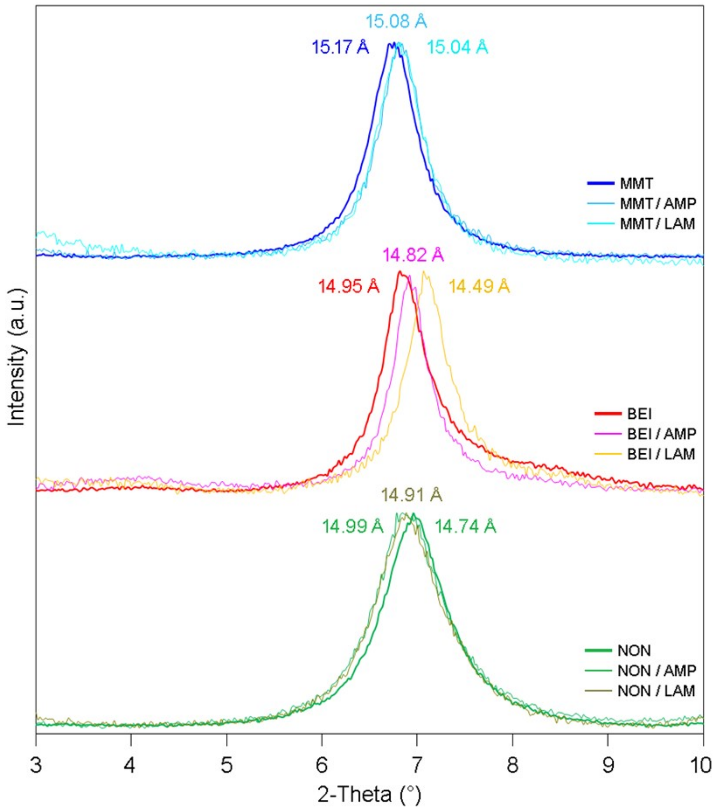

Fig. S9 Positions of basal reflections in XRPD patterns of original smectites before adsorption (MMT, BEI, NON) and after adsorption of AMP (MMT / AMP, BEI / AMP, NON / AMP) and LAM (MMT / LAM, BEI / LAM, NON / LAM). Additional information is available in Table S13.

Table S11

The highest experimentally determined equilibrium adsorption capacities for AMP or LAM on modified smectites (q_e (mod); in mg/g) and original smectites (q_e (orig); in mg/g). See also Figs. 3 and 6 in the manuscript. The last column (increase) gives the q_e (mod) / q_e (orig) ratios, i.e. how many times more AMP or LAM was adsorbed on the original smectite compared to the modified one.

drug	smectite	q_e (mod)	q_e (orig)	increase
AMP	MMT	24.85	47.01	1.89
AMP	BEI	5.18	53.07	10.24
AMP	NON	12.38	46.31	3.74
LAM	MMT	17.82	61.28	3.44
LAM	BEI	12.93	78.39	6.06
LAM	NON	19.60	46.98	2.40

Table S12

Freundlich, Langmuir and Toth adsorption isotherm parameters for the adsorption of each drug (AMP, LAM) onto each original smectite (MMT, BEI, NON) are listed. In addition to maximum adsorption capacities (q_m) and constants of adsorption isotherms (K_F, K_L, K_T, n), correlation coefficients (R^2) quantifying the match of a given isotherm with experimental data are also provided.

Freundlich	q_m	K_F	n	R^2
MMT / AMP	–	0.171	0.913	0.988
BEI / AMP	–	0.038	0.686	0.956
NON / AMP	–	0.081	0.821	0.957
MMT / LAM	–	3.384	1.604	0.827
BEI / LAM	–	68.833	16.029	0.435
NON / LAM	–	0.347	1.035	0.935
**Langmuir**	q_m	K_L	n	R^2
MMT / AMP	61090.298	$4.448 \cdot 10^{-6}$	–	0.984
BEI / AMP	156120.478	$2.096 \cdot 10^{-6}$	–	0.912
NON / AMP	104888.981	$2.243 \cdot 10^{-6}$	–	0.942
MMT / LAM	144.222	0.007	–	0.807
BEI / LAM	78.727	29.081	–	0.435
NON / LAM	876.208	$3.532 \cdot 10^{-4}$	–	0.935
**Toth**	q_m	K_T	n	R^2
MMT / AMP	50.654	0.005	570.549	0.981
BEI / AMP	44.185	0.007	$5.796 \cdot 10^{8}$	0.894
NON / AMP	40.817	0.006	4482.577	0.930
MMT / LAM	18.626	10.484	42.115	0.459
BEI / LAM	–	–	–	–
NON / LAM	50.531	0.006	22722.650	0.921

Table S13

For each original smectite before and after the adsorption of AMP or LAM, the position of the basal reflection maximum (2θ; in °2θ), the corresponding d_{001} value (d_{001}; in Å), the full width at half maximum of the basal reflection (FWHM; in °2θ) and the corresponding intervals of positions (2θ range; in °2θ) and d_{001} values (d_{001} range; in Å) are listed.

sample	2θ	d_{001}	FWHM	2θ range	d_{001} range
MMT	6.76	15.17	0.58	6.46–7.04	15.87–14.57
MMT / AMP	6.80	15.08	0.57	6.54–7.11	15.68–14.43
MMT / LAM	6.82	15.04	0.6	6.51–7.11	15.75–14.43
BEI	6.86	14.95	0.59	6.59–7.18	15.56–14.28
BEI / AMP	6.92	14.82	0.44	6.72–7.16	15.26–14.32
BEI / LAM	7.08	14.49	0.54	6.83–7.37	15.02–13.92
NON	6.96	14.74	0.77	6.58–7.35	15.59–13.95
NON / AMP	6.84	14.99	0.86	6.49–7.35	15.80–13.95
NON / LAM	6.88	14.91	0.82	6.51–7.33	15.75–13.99

Table S14

The numbers (N) of Na^+, H_2O, AMP and LAM molecules in models of the interlayer space of the original smectites with 1 AMP or 1 LAM and with no water. Corresponding d_{001} (Å) and E_{int} (kcal mol^{-1}; eqn (3)) values are also listed. The E_{int} values for the drug-Na^+ interaction ($E_{int,D-Na}$; kcal mol^{-1}; eqn (3b)) are provided in parentheses. Models with the lowest E_{int} values are displayed in Fig. S10 as indicated in the last column. With two exceptions in the case of AMP in NON (marked with asterisk), no d_{001} values fall within the d_{001} range determined from FWHM of the basal reflection of the corresponding real sample (see Table S13).

interlayer	N_{Na+}	N_{H2O}	N_{AMP}	N_{LAM}	d_{001}	E_{int} ($E_{int,D-Na}$)	Fig.
MMT	12	0	1	0	13.98	-90.85 (-32.15)	S10a
MMT	12	0	1	0	12.70	-90.74 (-27.54)	
MMT	12	0	1	0	13.12	-88.83 (-23.56)	
MMT	12	0	1	0	13.13	-79.00 (-15.62)	
MMT	12	0	1	0	16.07	-58.35 (-19.96)	
BEI	7	0	1	0	13.42	-96.21 (-31.85)	S10b
BEI	7	0	1	0	13.17	-95.09 (-26.39)	
BEI	7	0	1	0	13.24	-90.52 (-25.58)	
BEI	7	0	1	0	13.55	-78.99 (-14.13)	
BEI	7	0	1	0	18.94	-78.77 (-39.76)	
NON	15	0	1	0	12.93	-92.01 (-33.49)	S10c
NON	15	0	1	0	13.64	-72.84 (-17.01)	
NON	15	0	1	0	14.21*	-71.84 (-16.90)	
NON	15	0	1	0	15.14*	-65.54 (-16.78)	
NON	15	0	1	0	18.76	-58.82 (-20.46)	
MMT	12	0	0	1	11.01	-45.41 (-5.12)	S10d
MMT	12	0	0	1	10.73	-41.02 (-13.66)	
MMT	12	0	0	1	10.59	-39.70 (-6.06)	
MMT	12	0	0	1	10.74	-38.21 (-7.74)	
MMT	12	0	0	1	10.69	-36.62 (-7.53)	
BEI	7	0	0	1	11.00	-51.45 (-3.28)	S10e
BEI	7	0	0	1	10.92	-50.79 (-9.54)	
BEI	7	0	0	1	10.76	-50.58 (-9.56)	
BEI	7	0	0	1	10.84	-47.73 (-6.95)	
BEI	7	0	0	1	10.94	-47.55 (-5.86)	
NON	15	0	0	1	11.52	-49.95 (-3.00)	S10f
NON	15	0	0	1	11.28	-48.92 (-5.85)	
NON	15	0	0	1	11.13	-45.07 (-9.32)	
NON	15	0	0	1	11.40	-44.00 (-0.02)	
NON	15	0	0	1	11.09	-36.95 (-7.39)	

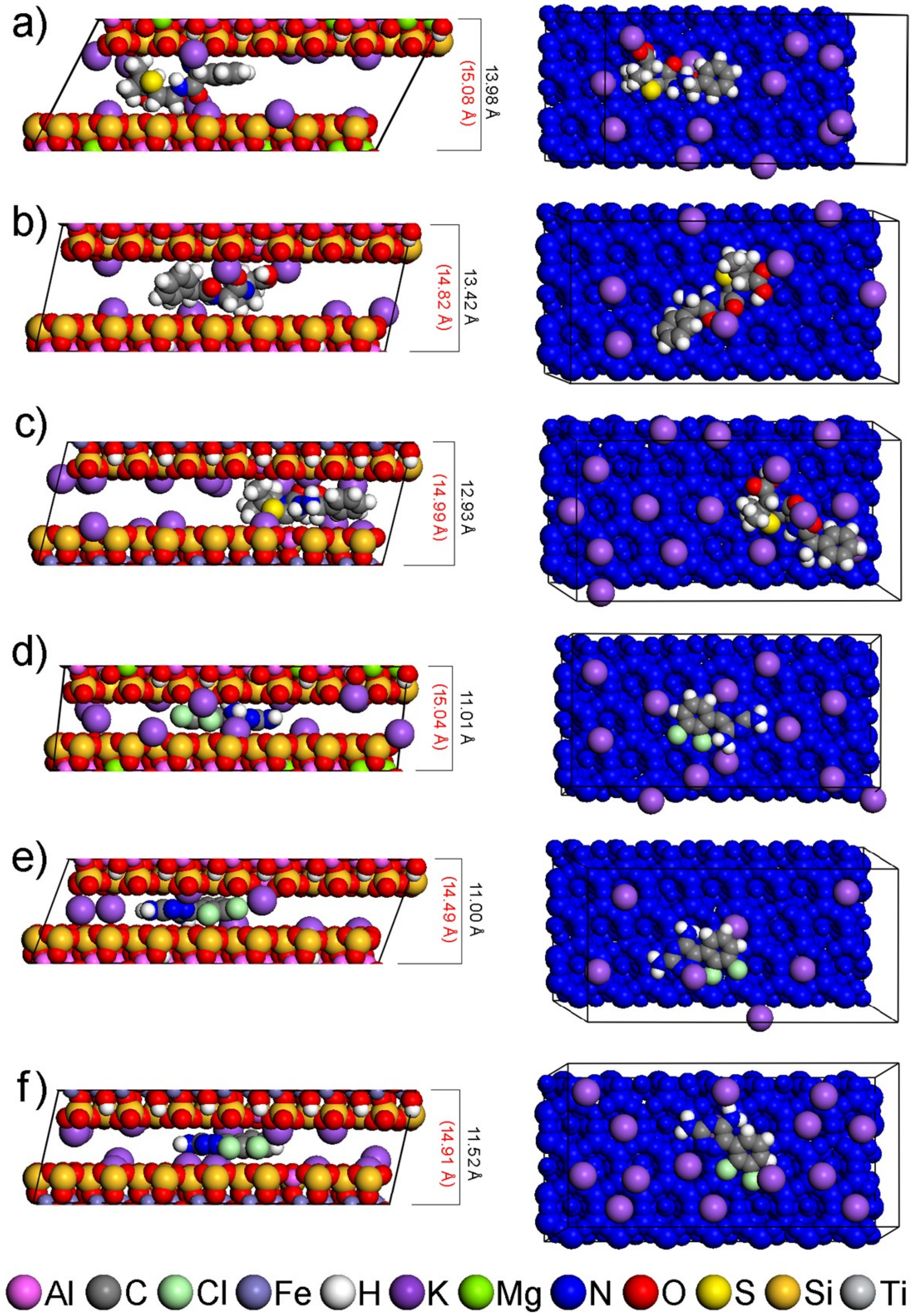

Fig. S10 Side views (left) and top views (right) of models of the interlayer space of original smectites with 1 drug molecule and with no water: (a) MMT / AMP, (b) BEI / AMP, (c) NON / AMP, (d) MMT / LAM, (e) BEI / LAM, (f) NON / LAM. The d_{001} values of the models and the experimental d_{001} values for comparison are written in black and red, respectively. For better clarity, each smectite is colored blue in the top views. Composition of the displayed models together with d_{001} and E_{int} values is available in Table S14.

Table S15

The numbers (N) of Na^+, H_2O, AMP and LAM molecules in models of the interlayer space of the original smectites with more AMPs or LAMs and with no water. Corresponding d_{001} (Å) and $E_{int,D(i)-sm}$ (kcal mol^{-1}; eqn (3d)) values are also listed. The E_{int} values for the drug-Na^+ ($E_{int,D(i)-Na}$; kcal mol^{-1}; eqn (3e)) and drug-drug ($E_{int,D(i)-D}$; kcal mol^{-1}; eqn (3c)) interaction are in parentheses and italics, respectively. Models exhibiting d_{001} values closest to the experimental d_{001} values are displayed in Fig. S11 as indicated in the last column. All d_{001} values belong to the d_{001} range determined from FWHM of the basal reflection of the corresponding real sample (see Table S13).

model	N_{Na+}	N_{H2O}	N_{AMP}	N_{LAM}	d_{001}	$E_{int,D(i)-sm}$ ($E_{int,D(i)-Na}$) $E_{int,D(i)-D}$	Fig.
MMT	12	0	2	0	14.47	-79.96 (-17.95); -79.05 (-18.83) *-4.58*	
MMT	12	0	2	0	13.96	-82.20 (-23.88); -78.58 (-14.19) *-0.47*	
MMT	12	0	2	0	14.28	-80.94 (-23.51); -61.28 (0.98) *-1.86*	
MMT	12	0	2	0	14.51	-69.36 (-14.64); -66.76 (-10.79) *-0.82*	S11a
BEI	7	0	7	0	14.96	-116.26 (-36.38) *-29.68*; -96.30 (-11.43) *-29.82*; -90.15 (-10.90) *-26.31*; -87.90 (-12.92) *-25.12*; -83.14 (-15.69) *-15.42*; -82.03 (-14.37) *-13.12*; -81.12 (-12.85) *-15.38*	S11b
NON	15	0	4	0	14.51	-86.21 (-25.96) *-8.92*; -79.78; (-10.36) *-18.52*; -79.19 (-22.69) *-8.00*; -68.10 (4.54) *-17.47*	S11c
MMT	12	0	0	3	14.07	-49.66 (-6.52) *-9.19*; -48.30 (-4.87) *-7.12*; -41.75 (1.98) *-3.30*	
MMT	12	0	0	3	14.44	-55.30 (-8.84) *-11.29*; -43.91 (-1.82) *-6.90*; -41.02 (-1.64) *-4.58*	S11d
BEI	7	0	0	6	14.82	-59.50 (-10.06) *-15.17*; -58.28 (-11.37); *-12.90*; -55.33 (-2.03) *-18.60*; -53.57 (-7.16) *-11.65*; -51.62 (-2.62) *-6.75*; -42.19 (-5.88) *-11.22*	S11e
NON	15	0	0	2	14.86	-41.30 (-0.42); -39.89 (-1.97) *-9.63*	
NON	15	0	0	2	14.83	-43.21 (-3.03); -41.46 (3.34) *-10.35*	
NON	15	0	0	2	14.89	-44.43 (0.04); -39.12 (2.39) *-10.89*	S11f

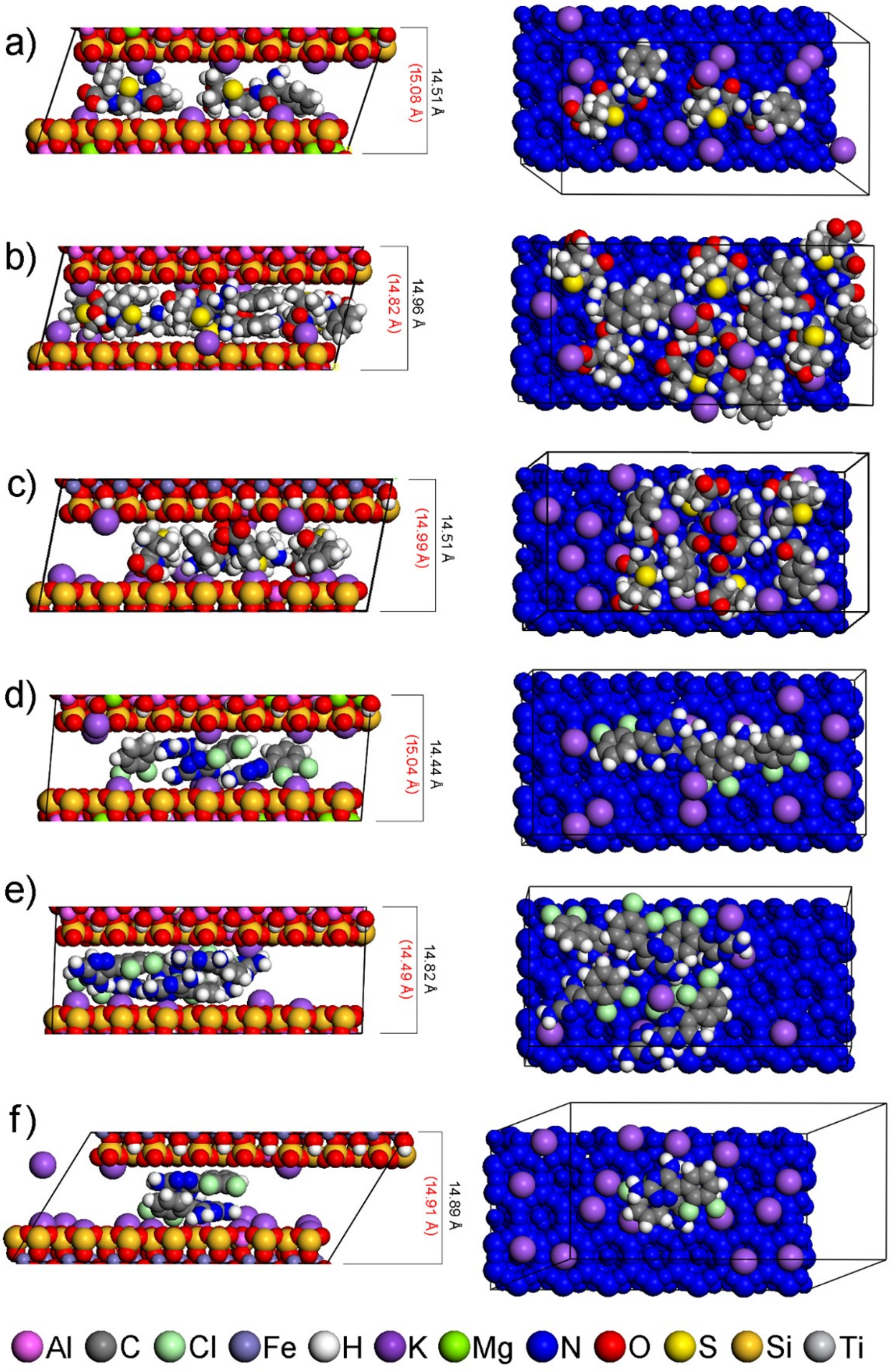

Fig. S11 Side views (left) and top views (right) of models of the interlayer space of original smectites with more drug molecules (i.e. 2, 7, 4 AMPs and 3, 6, 2 LAMs for MMT, BEI, NON, respectively) and with no water: (a) MMT / AMP, (b) BEI / AMP, (c) NON / AMP, (d) MMT / LAM, (e) BEI / LAM, (f) NON / LAM. The d_{001} values of the models and the experimental d_{001} values for comparison are written in black and red, respectively. For better clarity, each smectite is colored blue in the top views. Composition of the displayed models together with d_{001} and E_{int} values is available in Table S15.

Table S16

The numbers (N) of Na^+, H_2O, AMP and LAM molecules in models of the interlayer space of the original smectites with more AMPs or LAMs and with water. Corresponding d_{001} (Å) and $E_{int,D(i)-sm}$ (kcal mol^{-1}; eqn (3d)) values are also listed. The E_{int} values for the drug-Na^+ ($E_{int,D(i)-Na}$; kcal mol^{-1}; eqn (3e)) and drug-drug ($E_{int,D(i)-D}$; kcal mol^{-1}; eqn (3c)) interaction are in parentheses and italics, respectively. Models exhibiting d_{001} values closest to the experimental d_{001} values are displayed in Fig. 8 as indicated in the last column. All d_{001} values belong to the d_{001} range determined from FWHM of the basal reflection of the corresponding real sample (see Table S13).

model	N_{Na+}	N_{H2O}	N_{AMP}	N_{LAM}	d_{001}	$E_{int,D(i)-sm}$ ($E_{int,D(i)-Na}$) $\mathit{E_{int,D(i)-D}}$	Fig.
MMT	12	36	2	0	14.87	-75.86 (-2.52); -75.22 (-20.02) *-1.92*	
MMT	12	48	2	0	14.57	-82.68 (-20.19); -75.89 (-1.29) *-1.33*	
MMT	12	45	2	0	15.07	-79.48 (-19.92); -73.29 (-10.58) *-2.25*	8a
MMT	12	48	2	0	15.33	-78.62 (-20.33); -74.47 (-10.35) *-2.34*	
BEI	7	7	7	0	15.14	-98.12 (-30.51) *-17.84*; -96.45 (-29.13) *-17.04*; -89.36 (-25.38) *-15.17*; -85.65 (-2.24) *-23.91*; -83.77 (-14.87) *-21.94*; -80.68 (-16.06) *-16.84*; -77.13 (-8.53) *-24.07*	8b
NON	15	15	4	0	14.77	-91.15 (-27.57) *-11.71*; -85.90 (-28.88) *-10.04*; -83.05 (-16.82) *-13.58*; -75.67 (-6.45) *-12.69*	8c
NON	15	15	4	0	15.43	-82.79 (-28.06) *-9.97*; -82.64 (-24.71) *-12.56*; -63.81 (-3.27) *-7.12*; -59.83 (1.31) *-10.63*	
MMT	12	60	0	3	14.62	-55.35 (-2.76) *-5.04*; -54.14 (-5.21) *-10.81*; -41.85 (0.29) *-12.64*	
MMT	12	36	0	3	14.71	-53.27 (0.34) *-3.71*; -52.06 (-5.16) *-5.01*; -47.70 (0.57) *-4.93*	8d
BEI	7	14	0	6	14.58	-56.61 (-4.65) *-13.98*; -52.59 (-4.07) *-9.13*; -52.30 (-4.50) *-10.67*; -51.39 (-6.80) *-6.42*; -49.95 (-3.62) *-8.26*; -45.53 (1.79) *-6.83*	8e
NON	15	15	0	2	14.91	-49.39 (-6.48); -42.00 (2.15) *-10.59*	8f
NON	15	15	0	2	14.43	-49.54 (-3.07); -44.56 (-6.36) *-7.19*	
NON	15	30	0	2	14.65	-49.70 (-5.96); -47.67 (-0.33) *-9.04*	

Table S17

The numbers (N) of Na^+, AMP and LAM molecules in models of the surface of the original smectites. Corresponding E_{int} (kcal mol^{-1}; eqn (3)) values are also listed. Models with the lowest E_{int} values are displayed in Fig. S12 as indicated in the last column.

surface	N_{Na+}	N_{AMP}	N_{LAM}	E_{int}	Fig.
MMT	12	1	0	-38.98	S12a
MMT	12	1	0	-32.46	
MMT	12	1	0	-30.18	
MMT	12	1	0	-30.07	
MMT	12	1	0	-27.46	
BEI	7	1	0	-37.01	S12b
BEI	7	1	0	-33.61	
BEI	7	1	0	-32.87	
BEI	7	1	0	-29.87	
BEI	7	1	0	-29.19	
NON	15	1	0	-34.81	S12c
NON	15	1	0	-32.99	
NON	15	1	0	-29.47	
NON	15	1	0	-29.04	
NON	15	1	0	-25.82	
MMT	12	0	1	-28.36	S12d
MMT	12	0	1	-27.13	
MMT	12	0	1	-26.85	
MMT	12	0	1	-26.76	
MMT	12	0	1	-25.51	
BEI	7	0	1	-29.37	S12e
BEI	7	0	1	-29.16	
BEI	7	0	1	-28.41	
BEI	7	0	1	-28.27	
BEI	7	0	1	-27.03	
NON	15	0	1	-27.54	S12f
NON	15	0	1	-26.97	
NON	15	0	1	-26.93	
NON	15	0	1	-26.17	
NON	15	0	1	-25.11	

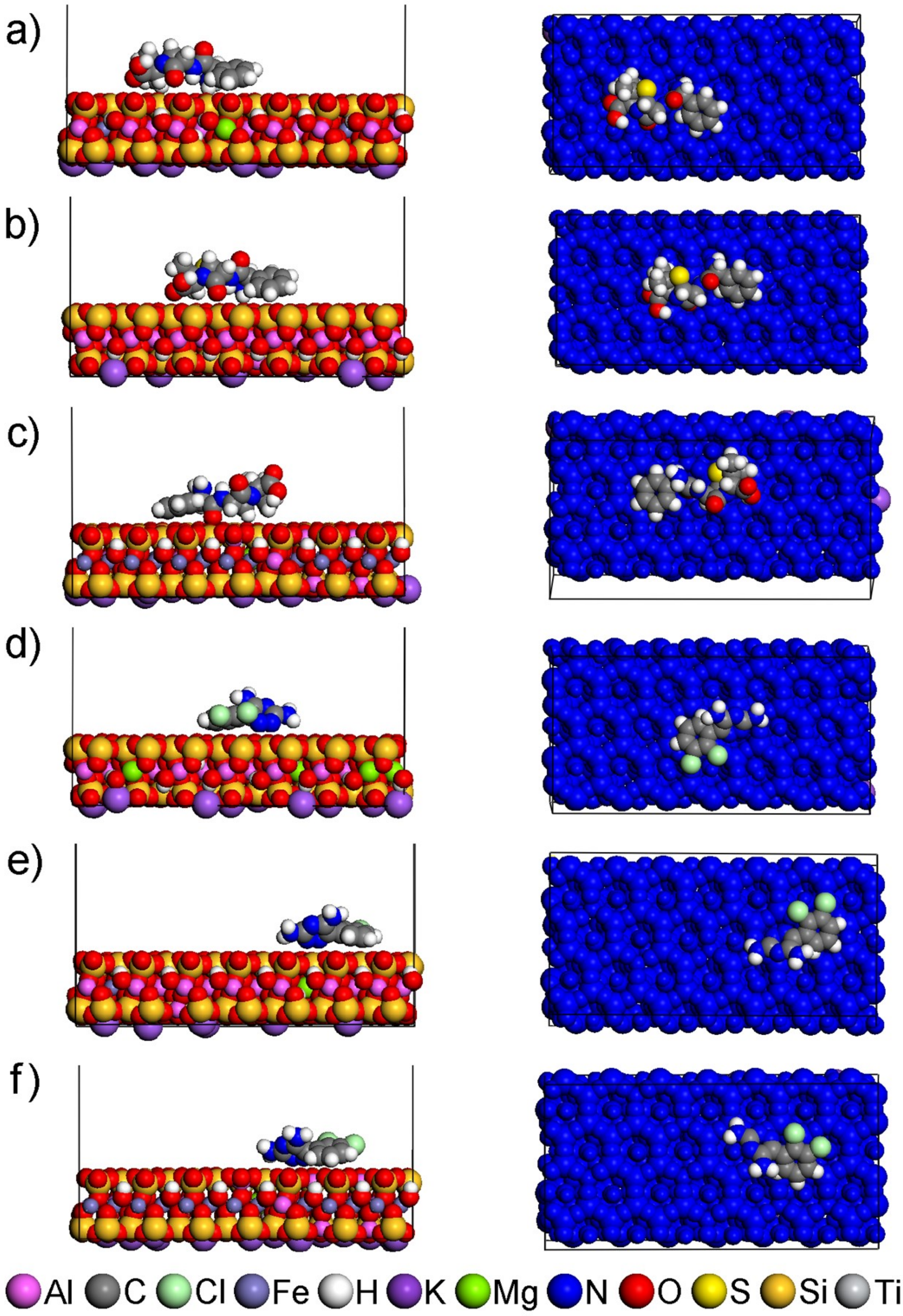

Fig. S12 Side views (left) and top views (right) of the models of the surfaces of original smectites with one drug molecule: (a) MMT / AMP, (b) BEI / AMP, (c) NON / AMP, (d) MMT / LAM, (e) BEI / LAM, (f) NON / LAM. For better clarity, each smectite is colored blue in the top views. Composition of the displayed models together with E_{int} values is available in Table S17.

Table S18

The numbers (N) of Na^+, AMP and LAM molecules in models of the surface of the original smectites. Corresponding $E_{int,D(i)-sm}$ (kcal mol^{-1}; eqn (3d)) values are also listed. The E_{int} values for the drug-drug interaction ($E_{int,D(i)-D}$; kcal mol^{-1}; eqn (3c)) are in italics. Models with the lowest average values of E_{int} are displayed in Fig. 9 as indicated in the last column.

surface	N_{Na+}	N_{AMP}	N_{LAM}	$E_{int,D(i)-sm}$ $E_{int,D(i)-D}$	Fig.
MMT	12	2	0	-43.46; -43.46 *-8.10*	9a
MMT	12	2	0	-44.71; 40.44 *-5.88*	
MMT	12	2	0	-42.88; -42.87 *-7.08*	
MMT	12	2	0	-40.45; -28.36 *-11.88*	
BEI	7	7	0	-65.42 *-27.37*; -54.59 *-29.50*; -54.13 *-18.89*; -49.23 *-19.63*; -40.23 *-19.27*; -21.40 *-20.05*; -19.58 *-18.07*	9b
NON	15	4	0	-57.14 *-29.51*; -48.02 *-18.41*; -45.30 *-15.21*; -24.85 *-23.49*	9c
NON	15	4	0	-58.50 *-28.40*; -53.10 *-16.72*; -33.88 *-14.94*; -23.45 *-21.86*	
MMT	12	0	4	-45.80 *-16.42*; -44.45 *-15.70*; -22.82 *-19.87*; -17.15 *-13.97*	9d
MMT	12	0	4	-43.18 *-16.49*; -35.48 *-9.11*; -17.02 *-13.41*; -16.49 *-13.95*	
BEI	7	0	6	-43.18 *-5.38*; -40.50 *-12.74*; -33.67 *-17.64*; -33.08 *-29.84*; -26.62 *-23.62*; -18.04 *-17.74*	9e
NON	15	0	2	-40.03; -15.84 *-13.52*	9f
NON	15	0	2	-37.14; -11.42 *-9.64*	
NON	15	0	2	-29.81; -27.79 *-1.85*	
NON	15	0	2	-29.58; -17.02 *-7.59*	

Table S19

Numerical values of c_0 (mg dm^{-3}), c_e (mg dm^{-3}), and q_e (mg g^{-1}) from the first (1) and second (2) adsorption experiment along with the ratios $q_{e(1)}/q_{e(2)}$ (%) and their average values (in bold; eqn (7)) quantifying the similarity of adsorptions. A visual comparison of q_e values from the first and the second adsorption experiment in dependence on c_0 values is provided in Fig. 10.

c_0	$c_{e(1)}$	$q_{e(1)}$	$c_{e(2)}$	$q_{e(2)}$	$q_{e(1)}/q_{e(2)}$	$c_{e(1)}$	$q_{e(1)}$	$c_{e(2)}$	$q_{e(2)}$	$q_{e(1)}/q_{e(2)}$
	MMT / AMP					MMT / LAM				
20	9.84	2.03	12.10	1.58	128.59	0.00	4.00	0.00	4.00	100.00
40	13.15	5.37	13.63	5.28	101.80	0.00	8.00	0.00	8.00	100.00
60	26.38	6.72	30.05	5.99	112.25	0.00	12.00	1.10	11.78	101.87
80	36.72	8.66	37.82	8.44	102.61	1.15	15.77	6.01	14.80	106.56
100	41.79	11.64	43.53	11.29	103.08	18.76	16.25	26.46	14.71	110.47
200	92.64	21.47	87.09	22.58	95.08	37.10	32.58	50.51	29.90	108.97
300	127.12	34.58	125.84	34.83	99.26	73.04	45.39	95.97	40.81	111.24
400	164.93	47.01	149.52	50.10	93.85	93.59	61.28	123.55	55.29	110.84
					**104.57±10.51**					**106.24±4.59**
	BEI / AMP					BEI / LAM				
20	12.59	1.48	12.04	1.59	93.10	0.00	4.00	0.00	4.00	100.00
40	22.04	3.59	26.54	2.69	133.43	0.00	8.00	0.00	8.00	100.00
60	22.35	7.53	26.92	6.62	113.83	0.00	12.00	0.00	12.00	100.00
80	34.32	9.14	33.44	9.31	98.12	0.00	16.00	0.00	16.00	100.00
100	42.69	11.46	43.92	11.22	102.20	0.00	20.00	0.00	20.00	100.00
200	90.27	21.95	70.00	26.00	84.40	0.00	40.00	0.13	39.97	100.07
300	117.77	36.45	109.72	38.06	95.77	0.11	59.98	5.98	58.80	102.00
400	134.63	53.07	113.98	57.20	92.78	8.04	78.39	15.30	76.94	101.89
					**101.70±14.38**					**100.49±0.84**
	NON / AMP					NON / LAM				
20	9.37	2.13	12.36	1.53	139.14	2.08	3.58	6.14	2.77	129.25
40	19.91	4.02	20.19	3.96	101.41	8.03	6.39	12.67	5.47	116.98
60	30.51	5.90	34.09	5.18	113.82	38.45	4.31	24.50	7.10	60.70
80	35.66	8.87	40.95	7.81	113.54	27.32	10.54	36.48	8.70	121.05
100	52.17	9.57	52.54	9.49	100.79	36.32	12.74	49.26	10.15	125.51
200	97.85	20.43	94.79	21.04	97.09	82.04	23.59	102.33	19.53	120.77
300	148.67	30.27	132.36	33.53	90.27	112.91	37.42	121.71	35.66	104.93
400	168.43	46.31	150.93	49.81	92.97	165.09	46.98	182.89	43.42	108.20
					**106.13±14.83**					**110.92±20.46**
